# School Nursing Guide for student health promotion: construction and validity

**DOI:** 10.1590/0034-7167-2022-0260

**Published:** 2022-12-16

**Authors:** Emanoel Avelar Muniz, Maria Veraci Oliveira Queiroz, Patrícia Neyva da Costa Pinheiro, Maria Rocineide Ferreira da Silva, Thereza Maria Magalhães Moreira, Eliany Nazaré Oliveira, Isabelline Freitas Dantas Paiva de Almeida, Valter Cordeiro Barbosa

**Affiliations:** I Instituto Federal de Educação, Ciência e Tecnologia do Ceará. Acaraú, Ceará, Brazil; II Universidade Estadual do Ceará. Fortaleza, Ceará, Brazil; III Universidade Federal do Ceará. Fortaleza, Ceará, Brazil; IV Universidade Estadual Vale do Acaraú. Sobral, Ceará, Brazil; V Instituto Federal de Educação, Ciência e Tecnologia do Ceará. Aracati, Ceará, Brazil

**Keywords:** Practice Guideline, School Nursing, Health Promotion, Educational Technology, Adolescent Health., Guía de Práctica Clínica, Servicios de Enfermería Escolar, Promoción de la Salud, Tecnología Educacional, Salud del Adolescente., Guia de Prática Clínica, Serviços de Enfermagem Escolar, Promoção da Saúde, Tecnologia Educacional, Saúde do Adolescente.

## Abstract

**Objectives:**

to describe the process of construction and validity of a School Nursing Guide for student health promotion.

**Methods:**

a methodological study, carried out from February to December 2021, composed of Convergent Care Research based on Pender’s Health Promotion Model. Based on the literature and dialogue with 11 nurses in the seven online focus groups, actions were constructed. Subsequently, 24 judges assessed content and appearance.

**Results:**

the guide proposes strategies for developing school nursing practices focusing on health promotion. The Appearance Validity Index ranged from 0.63 to 1.0, and the total was 0.84. The Content Validity Index ranged from 0.95 to 1.0, and the total was 0.997.

**Conclusions:**

the guide incorporated the needs of young people recognized by professionals, and the assessment phase confirms its validity, and can be used in the context of practice with young people.

## RESUMO

**Objetivos:**

descrever o processo de construção e validação do Guia de Enfermagem Escolar para promoção da saúde de jovens estudantes.

**Métodos:**

estudo metodológico, realizado de fevereiro a dezembro de 2021, composto por Pesquisa Convergente-Assistencial fundamentada no Modelo de Promoção da Saúde de Pender. A partir da literatura e diálogo com 11 enfermeiros nos sete grupos focais *online*, foram construídas ações. Posteriormente, 24 juízes avaliaram o conteúdo e a aparência.

**Resultados:**

o guia propõe estratégias para o desenvolvimento das práticas de enfermagem escolar enfocando a promoção da saúde. O Índice de Validade de Aparência variou de 0,63 a 1,0, e o total foi de 0,84. O Índice de Validade de Conteúdo variou de 0,95 a 1,0, e o total foi de 0,997.

**Conclusões:**

o guia incorporou as necessidades dos jovens reconhecidas pelos profissionais, e a fase de avaliação confirma sua validação, podendo ser utilizado no contexto da prática com jovens.

## RESUMEN

**Objetivos:**

describir el proceso de construcción y validación de la Guía Escolar de Enfermería para la promoción de la salud de los jóvenes estudiantes.

**Métodos:**

estudio metodológico, realizado de febrero a diciembre de 2021, compuesto por Investigación de Atención Convergente basada en el Modelo de Promoción de la Salud de Pender. Con base en la literatura y el diálogo con 11 enfermeras en los siete grupos focales en línea, se construyeron acciones. Posteriormente, 24 jueces evaluaron el contenido y la apariencia.

**Resultados:**

la guía propone estrategias para el desarrollo de prácticas escolares de enfermería con foco en la promoción de la salud. El índice de validez de apariencia osciló entre 0,63 y 1,0 y el total fue de 0,84. El índice de validez de contenido osciló entre 0,95 y 1,0 y el total fue de 0,997.

**Conclusiones:**

la guía incorporó las necesidades de los jóvenes reconocidas por los profesionales, y la fase de evaluación confirma su validación, pudiendo ser utilizada en el contexto de la práctica con jóvenes.

## INTRODUCTION

School nursing is a specialized practice responsible for intervening in students’ social, physical and emotional needs and developing comprehensive care, which favors academic success, lifelong achievement and students’ health^(1)^. Health care at school is a relevant mechanism for reaching vulnerable populations, especially young people, and has shown positive health outcomes. Challenges for these practices include workforce development, technology integration, financing and sustainability, and aligning health and education objectives^(2)^.

Research conducted internationally shows that school nurses intervene with students, parents/guardians and school staff, being associated with increased attendance, improved quality of schools and cost savings^(3)^. Its activities were classified into four main areas: (a) health promotion and disease prevention; (b) screening and treatment of acute issues; (c) management of chronic conditions; and (d) psychosocial support/support^(4)^.

A systematic review looked at the benefits of nursing’s role in health from a political and social point of view in countries where international level has been legally implemented. The thematic lines found were school public health policies, especially countries such as the United States, Spain and Mexico, with regulation on school nurses; on the other hand, in Latin America, there is a partial or non-existent regulation^(5)^.

In Brazil, there are political guidelines that include health actions in the school environment. Since 2007, the Health at School Program (PSE - Programa Saúde na Escola) and the Brazilian National Student Assistance Program (PNAES - Programa Nacional de Assistência Estudantil) stand out, starting in 2010. These programs have provided nursing professionals to take more directly health protection and promotion actions in educational environments. Although these programs include guidelines that articulate health and education policies, we identified weaknesses in intersectoral actions and, notably, in the role of nurses in this scenario; therefore, it is necessary to expand school nursing practices in the national context^(6)^.

A recently published World Health Organization (WHO) guideline on School Health Services (SHS) recommends that they should be comprehensively implemented, because rigorous evidence suggests that they are effective and acceptable. It offers a menu of 87 interventions, which were categorized into seven areas: health promotion; health education; screening; interventions preventive; clinical assessment; health service management; and support for other pillars of a health promoting school^(7)^.

From this perspective, school nurses need evidence-based Clinical Practice Guides to provide systematic and quality care to students. However, there is a scarcity of school nursing guides^(8)^, although there are some protocols and therapeutic guidelines to guide nurses’ practice in the care of students with needs and in specific clinical situations, such as drug administration^(9)^, use of epinephrine^(10)^, convulsion, epilepsy^(11)^ and asthma^(12)^. We identified only one guideline to increase physical activity in early childhood education schools up to 8th grade, which had the participation of a school nurse^(13)^. It is also noteworthy that all school nursing guides/guidelines found were produced in the United States.

Thus, to our knowledge, there is still no School Nursing Guide developed in a Latin American country focused on promoting young people’s health and also based on a nursing theory. In particular, Pender’s Health Promotion Model (HPM) proposes a framework to integrate nursing and behavioral science perspectives with factors that predict health behaviors^(14)^. The model guides how to explore biopsychosocial processes that motivate individuals to engage in behaviors that improve health and well-being, focusing on four main axes: physical activity, nutrition, stress management and social support^(14)^.

From this perspective, it is important to involve young people in improving their health condition, encouraging autonomy in choosing healthy behaviors. For the guide, the who proposed definition of young people was adopted, which uses the term for subjects aged 15 to 24 years^(15)^. Thus, understanding that school nursing is a field of action still strengthening in Latin American countries, such as Brazil, there is a need for instruments and technologies to support the development of actions in this area. Therefore, it is relevant to produce them based on scientific knowledge, in order to contribute to nursing science and its role in the theme of health promotion at school, strengthening care for adolescents and young people.

## OBJECTIVES

To describe the process of construction and validity of a School Nursing Guide to promote young students’ health.

## METHODS

### Ethical aspects

This study was approved by the Research Ethics Committees (REC) of the *Instituto Federal de Educação, Ciência e Tecnologia do Ceará* (IFCE) and the *Universidade Estadual do Ceará* (UECE). All ethical precepts for developing development this research were met, and participants’ consent was documented through the online Informed Consent Form (ICF).

### Study design, steps, period and location

The present study deals with development, testing of evidence of content and appearance validity, and assessment of methodological tools or strategies^(16)^. The research was carried out in two steps: School Nursing Guide participatory construction and testing of evidence of appearance, content and reliability validity with judges. Data were collected from February to December 2021.

### Step 1 - School Nursing Guide participatory construction

Initially, we performed an in-depth reading of Pender’s HPM^(14)^ and a survey of studies of nursing interventions in the school context, from an ongoing systematic review registered in the International Prospective Register of Systematic Reviews (PROSPERO), under number CRD42020178617. These studies were considered to extract the implemented and recommended strategies of school nursing focused on student health promotion. The strategies were thought and assessed in the following phases of a School Nursing Guide construction process.

In the guide construction, we adopted the Convergent Care Research (CCR) assumptions, a theorizing method on the problems that arise in practice, with a view to its resolution, or even provoking changes that contribute to qualifying assistance and introducing innovations for nursing and health care^(17)^. The CCR operationalization consists of 4 phases: conception, instrumentation, scanning and analysis^(17)^. At first, it started with the research problem (conception) formulation, decisions on the guide content construction (instrumentation) and the realization of focus groups (FG), such as moments of learning, sensitization, reading, reflection, discussion, in addition to agreements for changes shared with participants (peering and analysis)^(17)^.

For this step, IFCE nurses were invited to participate in the School Nursing Guide development. In this institution, professionals work at 33 *campi* throughout the state of Ceará, with technical students, integrated to high school, concomitant and subsequent, and higher education, with undergraduate and graduate courses, as well as initial and continuing education courses^(18)^. Therefore, the research was developed with professionals from this institution, due to the following reasons: 1) coverage of care to young students of different levels of education, sociocultural and economic contexts in the state; 2) institutional policy of student assistance involving the role of nurses in multidisciplinary teams within the school; 3) possibility for professionals to be involved in a collaborative process, i.e., active participation in guide discussion and construction, which would be of interest to the group.

During the study period, there were 28 nurses who were working in the Student Affairs Coordination (SAC) of IFCE *campi*, responsible for assisting young students. We included nurses who are working and effectively working in the IFCE SAC units, working for at least one year, suitable time to familiarize themselves with practices and establish a work routine, with interest and availability to participate in the research, actively collaborating in the guide development. We did not include nurses who were on leave or away from work. Finally, eleven nurses participated in this step of the research. They received an online form with questions about sociodemographic aspects, training and work, to characterize these participants, in addition to the ICF.

The FG was the technique used for the guide content participatory construction. Due to the COVID-19 pandemic, seven synchronous online FG^(19)^. Thus, communication between participants was simultaneous, from February to October 2021, through Google Meet, which allows the realization/recording of virtual conferences, as well as the exchange of messages through chat. The choice of virtual format was due to professionals being in remote work.

Nurses were invited in advance to participate in the FG, to define the best day, time and frequency of the meetings. The main researcher was the moderator, and experts on the subject were invited to collaborate with a dialogic presentation and discussion on the themes planned following the HPM. The meetings lasted about 2 hours, fortnightly periodicity, mostly, and had 4 to 10 participants. The FG recordings were analyzed by the principal researcher, who transcribed and extracted the main information. To preserve participants’ identity, they were identified with Arabic numerals in alphabetical order of names, such as Nur. 1, Nur. 2, and so on.

It is worth noting that at the last meeting there was the presentation and approval of the preliminary version of the School Nursing Guide, previously built by the main researcher based on the discussions of previous FG. The material was sent by e-mail to participants for reading, assessment and possible suggestions and comments 1 month in advance, and 2 reminders were made reinforcing the importance of reading the guide before the FG. At this time, the information was confirmed to make up the guide and necessary adjustments. In the analysis phase, the categorization process^(20)^ was constituted according to the guide theme organization, bringing contents and definitions of emerging actions in the discussions of FG.

The guide content was elaborated from the analysis of FG, HPM assumptions and literature consultation. Recommendations from the WHO Guideline for SHS^(7)^ and other health promotion guides already published in Brazil^(21-22)^ were also included, in addition to interventions from the Nursing Intervention Classification (NIC)^(23)^ and the International Classification for Nursing Practice (ICNP)^(24)^.

### Step 2 - Testing the guide evidence of appearance, content and reliability validity

After theoretical saturation, we tested the guide appearance and content validity, its reliability was assessed. Appearance validity is the aesthetic representation consisting of lines, shapes, colors and movement of images, which must harmonize with the information content^(25)^. And content validity constitutes a representation of a relevant sample of the universe of content that educational technology needs to contain^(26)^.

To assess guide, a committee of expert judges was made up. These are professionals working in the areas of health and/or education and with expertise in the area of care and/or health education with adolescents and young people in the school context and/or development and testing of evidence of validity of health technologies, preferably in protocols and clinical practice guides.

Those who achieved a minimum score of five points in the pre-defined criteria were selected: having a master’s and/or PhD degree with a dissertation/thesis in the area of interest; have specialization in public/collective health, child and adolescent health or pediatric nursing; have an article published in an indexed journal in the last 5 years on the theme; participate or coordinate research projects in the last 5 years on the theme; and/or have recent clinical practice of at least five years in the area of interest. The search for judges began with the identification of authors of publications on the theme of school health/nursing in Brazil, in addition to a search on the *Plataforma Lattes* of the Brazilian National Council for Scientific and Technological Development (CNPq - *Conselho Nacional de Desenvolvimento Científico e Tecnológico*) (https://lattes.cnpq.br /).

For the judges, an invitation was sent by e-mail in December 2021 explaining the objectives and procedures for assessing the technology appearance and content, in addition to a copy of the ICF, a copy of the guide, a form for a brief characterization of these judges, the Health Educational Technology Appearance Validity Instrument (HETAVI)^(25)^ and the Health Educational Content Validity Instrument (HECVI)^(26)^.

The HETAVI consists of 12 items on the style of illustrations, colors, shapes and distribution of figures on a 5-point Likert scale^(25)^. The HECVI consists of 18 items, divided into three domains: objectives (five), structure/presentation (ten) and relevance (three). Each item was assessed using a three-point Likert scale^(26)^. At the end of the instruments, an open-ended question was included for the judges to make possible suggestions/criticisms to improve the guide appearance and/or content. The suggestions were accepted, respecting their relevance and viability.

**Figure 1:**
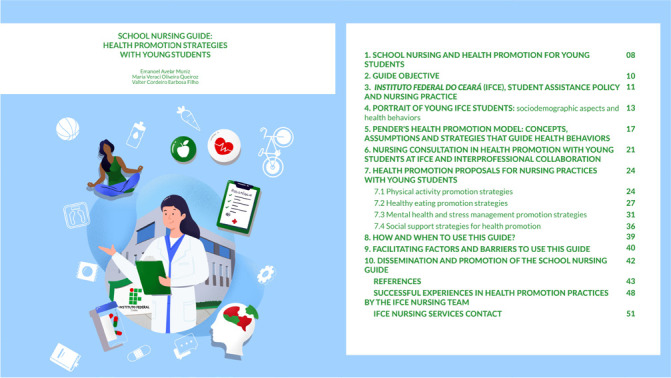
Cover and summary of the School Nursing Guide

To assess appearance, the Appearance Validity Index (AVI) was used. On a 5-point adjectival scale, the AVI for each item (I-AVI) was computed by the number of experts, who responded 4 or 5, divided by the total number of experts. For the total AVI (T-AVI), the sum of the I-AVI was performed, being divided by the total number of items. The item with AVI > 0.78 is considered excellent. The item with AVI between 0.60 and 0.77 indicates a need for suitability. The item with AVI < 0.60 is classified as bad, and the material must be redone from the key point of item^(25)^.

The CVI was calculated using the Item Content Validity Index (I-CVI) by content experts, who gave the item a relevance rating. The Total Content Validity Index (total-CVI) was calculated by the total/Ave-CVI (average) - average of the I-CVI and total-CVI/UA (universal agreement), which represents the proportion of items that achieved a rating of relevance by all experts^(16)^. It has been suggested that a CVI with a value of 0.90 or higher provides evidence of satisfactory content validity^(16)^.

Reliability in the technology equivalence dimension was assessed by the Intraclass Correlation Coefficient (ICC). The closer the value is to 1.0, the stronger the evidence of good reliability^(16)^. The binomial exact test was used to estimate the statistical reliability of AVI and CVI for each item, with a significance level of 5% (p>0.05).

## RESULTS

### School Nursing Guide development

The guide was constructed with the participation of eleven IFCE nurses. Their age ranged from 29 to 45 years (average of 34.2); 10 were female; 9 declared themselves white; 7 were married; the monthly gross family income ranged from R$ 5,300.00 (about US$963.63) to R$ 18,000.00 (about US$3,272.72) (average of R$ 10,344.00 ((about US$1,880.72)); 10 do not work in their municipality of origin; 7 have graduated for more than 10 years; and all have postgraduate degrees (4 specializations, 6 masters’ degree and 1 PhD degree). There were consultations with scientific literature, discussions and reflections on practice. It started from the guiding question: which theoretical and methodological aspects should make up a guide for nurses’ practice in health promotion in the school context with young people?

A synthesis of FG themes, nurses’ statements and decisions about the guide content are presented in Chart 1.

**Chart 1:** Synthesis of focus groups performed with nurses to construct the School Nursing Guide

Focus group	Themes discussed	Nurses’ statements	Incorporation into the guide
FG 1	- Presentation of the research project and Pender’s HPM; - Suggestions for changes in IFCE nurses’ practice.	*Seeking to understand students’ profile so that we can diagnose the situation and carry out actions focused on the needs of that moment. (Nur. 7)*	- Description of nursing consultation and successful experiences; - Definition of facilitating factors and barriers for carrying out and assessing health promotion actions.
FG2	- Analysis of health behaviors and associated factors of young IFCE students.	*[...] mental health is one of the biggest complaints that reach us [...] there should be more actions focused on mental health!* (Nur. 10)	- Portrait of young IFCE students; - Strategies for mental health promotion and stress management.
FG3	Healthy eating promotion in the school context with young people: possibilities for interventions.	*The day the company [food] takes a salad is the day that students less go to line up and still complain! [...].* (Nur. 2)	- Encouraging the consumption of natural and organic foods; - Resistance of students to acquire/maintain healthy eating behaviors.
FG4	Physical activity promotion in the school context with young people: possibilities of interventions.	*The games are very present in my campus, whenever there are games, we have a very large audience that wants to go!* (Nur. 2)	- Facilitating factors and barriers to physical activity promotion in IFCE; - Continuous support/encouragement to students in physical activity practices.
FG5	Mental health and stress management intervention promotion with young people in the school context.	*With auriculotherapy, I had more visibility [...] these practices [ICP] give quick answers!* (Nur. 7)	- Facilitating factors and barriers in the approach to young people’s mental health; - Use of art and Integrative and Complementary Practices.
FG6	Social support and health promotion: possibilities of interventions in the school context.	*We always fight with the teaching sector to ensure a space in the school calendar for actions!* (Nur. 5)	- Integration with the other sectors of the *campus*, conducting home visits and strengthening the bond of students with the institution.
FG7	Presentation and approval of the preliminary version of the School Nursing Guide.	*The guide is a way for us to record and systematize what we already do and also expand our strategies*. (Nur. 1)	- Limits and possibilities of nursing professionals’ performance in school health and consensual adjustments in technology.

*HPM - Health Promotion Model; IFCE - Instituto Federal de Educação, Ciência e Tecnologia do Ceará.*

The guide aims to guide IFCE nursing professionals’ practice focusing on health promotion strategies and care for young people; however, it can also be used by other nursing and health professionals who work directly with young people, either in educational institutions or in Primary Health Care. It was organized contemplating theoretical and practical aspects presented in the sequence: introduction to school nursing, its potential and challenges in promoting students’ and guide objective. Then, the institutional context of IFCE, the Student Assistance Policy and the role of nursing in that policy were introduced. A portrait of the socioeconomic aspects and health situation of young IFCE students was added, obtained through a cross-sectional study.

Furthermore, essential elements of Pender’s HPM were brought. The nursing consultation was briefly presented, held with IFCE young students, indicating proposals for health promotion to be inserted in the care of students. The strategies and respective actions of health promotion proposed in the development of school nursing practices stand out. To promote physical activity, there are 25 proposals for actions. In healthy eating promotion, there are 19 proposals for actions. In mental health and stress management promotion, 22 actions are proposed, and in the strategies of social support for health promotion, there are 17 proposals for actions. Finally, some factors that may appear as facilitators or barriers to use the guide in clinical and educational practice are presented, as well as some actions to disseminate and disseminate the knowledge produced by the guide, in addition to successful experiences in health promotion practices developed by IFCE nursing and student assistance teams.

The guide was sent only once to the participating nurses and expert judges before the final version was approved, and three researchers were more directly involved in the guide appearance and content elaboration and organization.

### Guide validity evidence of appearance, content and reliability

The 24 judges who assessed the guide were 29 to 57 years old (average of 39.2 years), 83.3% of them female. Thus, 19 were nurses, 2 were psychologists, 1 were physical education professionals, 1 was a pedagogue and 1 was a designer. These professionals were linked to 15 institutions in 9 states of the 5 regions of Brazil and 50% held a PhD degree; of these, 58.3% had thesis in the area of interest. All had a master’s degree, 50% with dissertation in the area of interest; 25% had specialization in public/collective health, child and adolescent health and/or pediatric nursing; 54.2% had an article published in the last 5 years on the subject; 83.3% participated in or coordinated research projects in the last 5 years on the subject; and 50% had recent clinical practice of at least five years as school nurses.

The expert judges responded to HETAVI. Table 1 shows the answers to each question asked, in addition to AVI for each item.

**Table 1:** Expert judges’ assessment (n=24) of the School Nursing Guide appearance through the Health Educational Technology Appearance Validity Instrument, Fortaleza, Ceará, Brazil, 2022

Items	1	2	3	4	5	AVI^¶^	*p* value
	TD^^*^^	D^†^	PD^‡^	A ^§^	TA^||^		
1. Illustrations are suitable for the target audience	1	0	3	12	8	0.83	0.460
2. Illustrations are clear and convey ease of understanding	0	0	5	12	7	0.79	0.540
3. Illustrations are relevant to the understanding content by the target audience	0	0	2	14	8	0.92	0.115
4. Illustrations’ colors are suitable for the type of material	0	0	1	10	13	0.96	0.033^^**^^
5. Illustrations’ shapes are suitable for the type of material	0	0	4	11	9	0.83	0.460
6. Illustrations depict the daily life of the intervention’s target audience	1	0	3	7	13	0.83	0.460
7. Figure arrangement is in harmony with the text	0	0	3	8	13	0.87	0.264
8. The figures used elucidate the educational material content	1	0	2	15	6	0.87	0.264
9. Illustrations help in exposing the theme and are in a logical sequence	0	0	0	15	9	1.0	0.091
10. Illustrations are in suitable quantity in the educational material	0	0	6	10	8	0.75	0.540
11. Illustrations are in suitable size in the educational material	0	1	2	13	8	0.87	0.149
12. Illustrations help in changing behaviors and attitudes of the target audience	2	0	7	11	4	0.62	0.000^^**^^
Total						0.84	

* TD - totally disagree;

† D - disagree;

‡ PD - partially disagree;

§ A - agree;

|| TA - totally agree; ¶AVI - Appearance Validity Index; p-value - binomial exact test (a - alternative hypothesis states that the proportion of cases in the first group <0.80);

** Statistically significant disagreement (p<0.05).

I-AVI ranged from 0.62 to 1.0, and total-AVI was 0.84, which can be considered good. Only 2 items (10 and 12) presented <0.78 and were considered to improve the guide appearance. The ICC obtained was 0.91, evidencing excellent reliability in the guide appearance assessment by judges. According to the binomial exact test, there was significant disagreement (p<0.05) between judges in the scores for items 4 and 12, which refer to illustration colors and the support of these illustrations in changing behavior.

The judges suggested increasing the number of figures that represent the text content and that, preferably, were copyrighted. In addition to improving the representation of black students and teenagers in school uniform, including the image of a nurse on the guide cover and reviewing some graphic elements that referred to the hospital context, considering it to be unnecessary.

The expert judges also answered the HECVI. Table 2 below shows the answers for each question, the domains, in addition to the CVI - I and the CVI Total/Ave obtained.

**Table 2:** Expert judges’ assessment (n=24) of the School Nursing Guide content through the Health Education Content Validity Instrument, Fortaleza, Ceará, Brazil, 2021

Item	D^*^	PA^†^	TA^‡^	CVI^§^	*p* value
OBJECTIVES	0	1	2	1.0	
1. Contemplates proposed theme	0	3	21	1.0	0.661
2. Suitable for the teaching-learning process	0	4	20	1.0	0.661
3. Clarifies doubts about the theme covered	0	5	19	1.0	0.661
4. Provides reflection on the theme	0	5	19	1.0	0.661
5. Encourages behavior change	0	5	19	1.0	0.661
STRUCTURE/PRESENTATION	0	1	2	0.99	
6. Language suitable for the target public	0	2	22	1.0	0.661
7. Language appropriate to educational material	0	3	21	1.0	0.661
8. Interactive language	0	11	13	1.0	0.661
9. Correct information	0	2	22	1.0	0.661
10. Objective information	0	6	18	1.0	0.661
11. Enlightening information	0	3	21	1.0	0.661
12. Necessary information	0	3	21	1.0	0.661
13. Logical sequence of ideas	0	2	22	1.0	0.661
14. Current theme	0	2	22	1.0	0.661
15. Appropriate text size	1	7	16	0.96	0.292
RELEVANCE	0	1	2	1.0	
16. Encourages learning	0	3	21	1.0	0.661
17. Contributes to knowledge in the field	0	0	24	1.0	0.661
18. Arouses interest in the theme	0	2	22	1.0	0.661
Total/Ave- CVI				0.998	

* D - disagree;

† PA - partially agree;

‡ TA - totally agree; ^**§**^ *Content Validity Index; p-value - exact binomial test (a - states of alternative hypotheses than the proportion of cases in the first group <0.80).*

The HECVI results showed a I-CVI ranging from 0.96 to 1.0. The total/Ave-CVI of 0.998 and the total-CVI/UA of 0.94. There was no significant disagreement between judges (p>0.05) in the item scores and the ICC obtained was 0.73, which represents moderate reliability in the content assessment. These data demonstrate that the guide has excellent evidence of content validity.

Considering judges’ suggestions to improve the guide appearance and content, in the final version, illustrations that were not in good resolution were replaced, increasing the number of illustrations, mainly authorial, that portray students’ and professionals’ daily lives. Regarding content, some excerpts were reduced, mainly from the theoretical framework, relating to the nursing consultation and health promotion strategies. This includes a brief discussion of the most common interprofessional practices and mental health problems among young students.

## DISCUSSION

The present study elaborated and tested evidence of validity of a School Nursing Guide that provides structured information on health promotion strategies that can be developed with young students. In this sense, the use of educational technologies for nursing professional development is an important component in creating effective, convenient and accessible education for professionals, managers and users in different healthcare systems and geographic locations^(27)^.

As a field of action, school nursing is strengthened with the production of educational technology, using methodological rigor, guided by CCR and other theoretical foundations that are essential in nursing practice. Furthermore, creating content and testing the validity of a practice guide contributes to the introduction of a technological innovation in the work process of professionals in a specific area of expertise^(28)^.

In health care, nursing theories are able to bring benefits to their ability to produce consistent explanations, descriptions, predictions and prescriptions, which support the profession in the performance in complex contexts^(29)^. In this sense, HPM and CCR enabled a (re)interpretation of reality and a better understanding of the factors related to the health behaviors of young students (barriers and perceived benefits), as well as the construction of interventions in a shared way.

It is noteworthy that the process of changes and/or innovations in nursing and health care is characterized as a collective work. Thus, the researcher needs to negotiate the change/innovation project with the social actors working in the context, seeking their commitment. This negotiation requires time, patience, persistence and, above all, a viable proposal, well elaborated and with accessible language^(17)^. Thus, nurses’ involvement as an interested party in the guide development and dissemination in care practice occurred both in the negotiation to carry out the research and in the definition of its object and discussion of proposed health promotion strategies.

The guide as an advisory tool provides structured information on health promotion strategies that can be developed with young students. Thus, nurses can seek information for decision-making, according to the demand of their care practice, in one or more of these strategies, considering their expertise, the educational institution’s conditions and students’ needs so that they have an active role in the pursuit of health and self-care. It is important to consider the specificities in the approach to young people, such as age group (adolescents aged between 15 and 19 years or older youths aged between 20 and 24 years), in addition to sociocultural differences, gender, sexual orientation, among others, to direct and individualize care.

Thus, understanding the various factors involving the young person’s life makes the term “youth” require pluralization, thus using “youths” to determine various realities found in these youth groups. When referring to youth, the meeting of these young people in their different scenarios of existence allows nurses to screen for diagnoses and interventions aimed at the needs of their own age group, taking into account their reality and providing clinical care^(30)^.

Regarding the guide appearance and content assessment process, it is believed that experts’ point of view motivated the review of these aspects, incorporating suggestions to make the material more interactive and suitable to nurses’ needs and practices in the school context. It was also of paramount importance the collaboration of graphic designer professionals in the guide layout and illustration elaboration. According to the results, the technology internal validity is confirmed, which suggests the possibility of advancing to the next steps with intervention research on the effectiveness of its implementation in the field of practice.

The suggestions pointed out by expert judges in the steps of content and appearance validity indicate the importance of submitting educational devices to validity processes^(31)^. It is also noteworthy the incorporation of educational materials based on nursing theories, submitted to validity processes to mediate health education practices, and students’ awareness, with a view to approaching and dialoguing with youth.

Given the above, having been built from the CCR is one of the strengths of the guide, considering the experiences of researchers in their workplace, which enriches its content and feasibility, presenting what actually works in that educational environment. The guide is a form of translation from scientific knowledge to practice. However, several challenges are involved in its implementation. It is crucial to have an appropriate strategy to ensure that the necessary structures, material and human resources are available. Including school nurses in the development and implementation of health promotion strategies is vital for improving young students’ living conditions and health.

### Study limitations

In the guide, only the four health promotion domains proposed by HPM were addressed, which are physical activity, healthy eating, stress management and social support. However, there are other themes related to health promotion that can be developed by nurses in the school context, such as sexual and reproductive health, substance use, chronic diseases, among others, because they are necessary to this population. Another limitation of this study is the fact that it did not return the version of the validated guide with judges’ suggestions to the professionals who collaborated in the construction, because it is about the development of a technology in a participatory way, with recommendations for it to be carried out later.

### Contributions to nursing, health, and public policies

The guide was built in the context of IFCE nurses’ practice; however, it is believed that the proposed strategies can be adapted and used in other educational institutions and by Family Health Strategy nurses in PSE actions. Through this technology, it is possible to plan and develop actions that contribute to change health behaviors of young students, strengthening their autonomy and responsibility, collaborating in health promotion.

## CONCLUSIONS

The School Nursing Guide was constructed based on students’ needs, recognized in other studies and in the experiences of IFCE nurses, with HPM as a framework. It is considered that the guide has evidence of validity, bringing contributions to good school nursing practices, encouraging innovation in care by involving nurses’ educational practice. The development and/or improvement of health promotion strategies with young students, proposed by the guide, therefore envisage changes in practices, reverberating with the strengthening of autonomy and empowerment of young people in promoting their health.

## SUPPLEMENTARY MATERIAL

https://ifce.edu.br/noticias/noticias-de-destaque/201cguia-de-enfermagem-escolar201d-trazdiagnostico-e-estrategias/guia-de-enfermagem-escolar-1.pdf/view.

0034-7167-reben-76-01-e20220260-sup01

## References

[1] Maughan ED, Jameson BE. (2020). Celebrating 21st-Century School Nursing Practice. NASN Sch Nurse.

[2] North S, Dooley DG. (2020). School-Based Health Care. Prim Care Clin Office Pract.

[3] Best NC, Oppewal S, Travers D. (2018). Exploring school nurse interventions and health and education outcomes: an integrative review. J Sch Nurs.

[4] Lineberry MJ, Ickes MJ. (2015). The role and impact of nurses in American elementary schools: a systematic review of the research. J Sch Nurs.

[5] Pérez MFC, Castillo NIC, Vergara CFS, Saavedra AFB, Flores RIV. (2021). Beneficios del rol de enfermería en salud escolar, implementación a nivel internacional: revisión sistemática. Horizonte Enferm.

[6] Muniz EA, Queiroz MVO, Dutra FCS, Araújo AF, Silva LMS, Torres RAM. (2021). Políticas de saúde e educação para a juventude no brasil: intersetorialidade e atuação do enfermeiro. Sanare (Sobral, Online).

[7] World Health Organization (WHO) (2021). WHO Guideline on School Health Services.

[8] Shannon RA, Maughan ED (2020). A model for developing evidence-based clinical practice guidelines for school nursing. J Sch Nurs.

[9] Bergren MD. (2022). NASN’s Medication Administration Clinical Guideline. NASN Sch Nurse.

[10] Tanner A, Clarke C. (2016). Epinephrine Policies and Protocols Guidance for Schools: Equipping School Nurses to Save Lives. NASN Sch Nurse.

[11] Lepkowski AM, Maughan ED. (2018). Introducing NASN’s New Evidence-based Clinical Guideline: students with seizures and epilepsy. NASN Sch Nurse.

[12] Maughan ED, Schantz S. (2014). NASN’s first evidence-based clinical guidelines: asthma. NASN Sch Nurse.

[13] Bagby K, Adams S. (2007). Evidence-based practice guideline: increasing physical activity in schools--kindergarten through 8th grade. J Sch Nurs.

[14] Murdaugh CL, Parsons MA, Pender NJ. (2019). Health Promotion in Nursing Practice.

[15] World Health Organization (WHO) (1986). Report of a WHO Study Group on Young People and Health for All. Technical Report Series 731.

[16] Polit DE, Beck CT. (2019). Fundamentos de pesquisa em enfermagem: avaliação de evidências para a prática de enfermagem.

[17] Trentini M, Paim L, Silva DMG. (2014). Pesquisa convergente-assistencial: delineamento provocador de mudanças nas práticas de saúde.

[18] Instituto Federal do Ceará (2017). Pró-reitoria de Ensino. Plano Estratégico para Permanência e Êxito dos Estudantes do IFCE.

[19] Salvador PTCO, Alves KYA, Rodrigues CCFM, Oliveira LV. (2020). Online data collection strategies used in qualitative research of the health field: a scoping review. Rev Gaúcha Enferm.

[20] Gibbs G. (2009). Análise de dados qualitativos.

[21] Ministério da Saúde (BR) (2021). Departamento de Promoção da Saúde. Guia de Atividade Física para a População Brasileira.

[22] Ministério da Saúde (BR) (2014). Departamento de Atenção Básica. Guia alimentar para a população brasileira.

[23] Bulechek GM, Butcher HK, Dochterman JM, Wagner CM. (2016). Classificação das Intervenções em Enfermagem (NIC).

[24] International Council of Nurses (2017). Classificação Internacional para a Prática de Enfermagem (CIPE®) -Português do Brasil [Internet].

[25] Souza ACC, Moreira TMM, Borges JWP. (2020). Development of an appearance validity instrument for educational technology in health. Rev Bras Enferm.

[26] Leite SS, Áfio ACE, Carvalho LV, Silva JM, Almeida PC, Pagliuca LMF. (2018). Construction and validation of an Educational Content Validation Instrument in Health. Rev Bras Enferm.

[27] Weinschreider J, Sabourin KM, Smith CM. (2019). Preparing Nurse Leaders in Nursing Professional Development. J Nurses Prof Dev.

[28] Zanon BP, Paula CC, Ribeiro AC, Padoin SMM. (2022). Content validation to support the monitoring of disclosure of HIV diagnosis in childhood. Rev Bras Enferm.

[29] Brandão MAG, Barros ALBL, Primo CC, Bispo GS, Lopes ROP. (2019). Nursing theories in the conceptual expansion of nursing practices. Rev Bras Enferm.

[30] Castro AR, Santos MAP, Ribeiro FCS, Santos IC, Felício JR, Silva MRF. (2019). Habitando territórios: construções e desconstruções na educação em saúde sobre a sexualidade junto a adolescentes. Braz J Health Rev.

[31] Gigante VCG, Oliveira RC, Ferreira DS, Teixeira E, Monteiro WF, Martins ALO (2021). Construction and validation of educational technology about alcohol consumption among university students. Cogitare Enferm.

