# Supplementary material for: School Nursing Guide for student health promotion: construction and validity
Source: Rev Bras Enferm. 2022 Dec 16;76(1):e20220260. doi: 10.1590/0034-7167-2022-0260 (PMC9749768; doi:10.1590/0034-7167-2022-0260)
Supplement: 0034-7167-reben-76-01-e20220260-sup01 [file 0034-7167-reben-76-01-e20220260-sup01.pdf]

GUIA DE ENFERMAGEM ESCOLAR: ESTRATÉGIAS DE PROMOÇÃO DA SAÚDE COM JOVENS ESTUDANTES

Emanoel Avelar Muniz
Maria Veraci Oliveira Queiroz
Valter Cordeiro Barbosa Filho

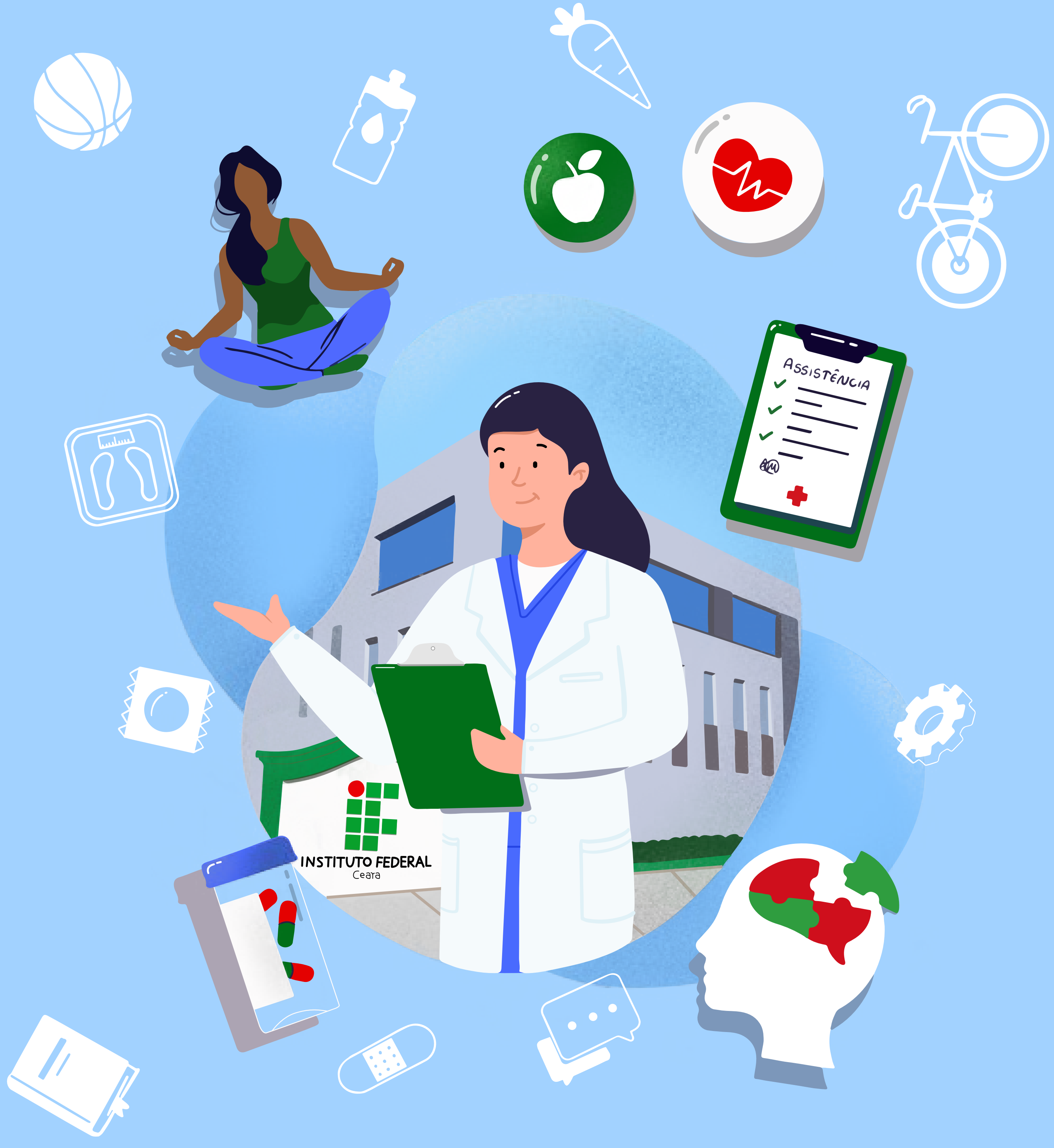

INSTITUTO FEDERAL DE EDUCAÇÃO, CIÊNCIA E TECNOLOGIA
DO CEARÁ -IFCE

DIRETORIA DE ASSUNTOS ESTUDANTIS - DAE

SERVIÇO DE ENFERMAGEM

GUIA DE ENFERMAGEM ESCOLAR: ESTRATÉGIAS DE PROMOÇÃO DA SAÚDE COM JOVENS ESTUDANTES

FORTALEZA
2022

EXPEDIENTE

Reitor do Instituto Federal de Educação, Ciência e Tecnologia do Ceará
Jose Wally Mendonça Menezes

Pró-Reitora de Ensino
Cristiane Borges Braga

Pró-Reitora de Extensão
Ana Cláudia Uchôa Araújo

Pró-Reitora de Pesquisa, Pós-Graduação e Inovação
Joélia Marques de Carvalho

Pró-Reitor de Administração e Planejamento
Reuber Saraiva de Santiago

Pró-Reitor de Gestão de Pessoas
Marcel Ribeiro Mendonça

Diretora de Assuntos Estudantis
Ana Caroline Cabral Cristino

Dados Internacionais de Catalogação na Publicação
Instituto Federal de Educação, Ciência e Tecnologia do Ceará
Sistema de Bibliotecas – SIBI

I59g Instituto Federal do Ceará. Reitoria. Diretoria de Assuntos Estudantis. Serviço de Enfermagem
Guia de enfermagem escolar: estratégias de promoção da saúde com jovens estudantes / Organização Emanoel Avelar Muniz; Maria Veraci Oliveira Queiroz; Valter Cordeiro Barbosa Filho. – Fortaleza: IFCE, 2022.
54 p. il. color.

ISBN: 978-65-87470-39-9 (livro digital) 392 MB

1. Instituto Federal do Ceará – Enfermagem escolar. 2. Instituto Federal do Ceará – Assistência estudantil. 2. IFCE – Promoção da saúde estudantil. I. Muniz, Emanoel Avelar (Org.). II. Queiroz, Maria Veraci Oliveira (Org.). III. Barbosa Filho, Valter Cordeiro (Org.). IV. IFCE/ Reitoria/ Diretoria de Assuntos Estudantis. V. Título.

CDD (20.ed.) 610.73

Bibliotecária responsável: Etelvina Maria Marques Moreira CRB N° 3/615

ORGANIZAÇÃO

Emanoel Avelar Muniz
Enfermeiro do IFCE – *Campus* Acaraú e Doutorando em Cuidados Clínicos em Enfermagem e Saúde pela Universidade Estadual do Ceará (UECE).

Maria Veraci Oliveira Queiroz
Enfermeira, Doutora em Enfermagem pela Universidade Federal do Ceará (UFC), docente do Programa de Pós-graduação Cuidados Clínicos em Enfermagem e Saúde (PPCCLIS) e do Curso de Graduação em Enfermagem da UECE.

Valter Cordeiro Barbosa Filho
Professor de Educação Física do IFCE – *Campus* Aracati, Doutor em Educação Física pela Universidade Federal de Santa Catarina (UFSC) e docente do Programa de Pós-graduação em Saúde Coletiva (PPSAC) da UECE.

COLABORAÇÃO

Carla Lidiany Bezerra S. Oliveira – Enfermeira do IFCE – *Campus* Limoeiro do Norte
Denise Tomaz Aguiar – Enfermeira do IFCE – *Campus* Quixadá
Edmara Teixeira Oliveira – Enfermeira do IFCE – *Campus* Canindé
Fabiani Weiss Pereira – Enfermeira do IFCE – *Campus* Avançado de Jaguaruana
Ítala Keane Rodrigues Dias – Enfermeira do IFCE – *Campus* Iguatu
Jadna Mony Gregorio Freitas – Enfermeira do IFCE – *Campus* Cedro
Kaline Ribeiro de Freitas – Enfermeira do IFCE – *Campus* Morada Nova
Marcela Lima Silveira Praxedes – Enfermeira do IFCE – *Campus* Aracati
Milena Leite Albano - Enfermeira do IFCE – *Campus* Paracuru
Paulo César Teles de Almeida – Enfermeiro do IFCE – *Campus* Itapipoca
Sharlene Pereira Alves - Enfermeira do IFCE – *Campus* Tauá

REVISÃO ORTOGRÁFICA
Carmela Carolina Alves de Carvalho

EDIÇÃO E DIAGRAMAÇÃO
Fabiele do Amaral Beck

ILUSTRAÇÕES
Elita Maria Ramos Colares Ferreira

APOIO
Equipe de Enfermagem do IFCE
Programa de Pós-graduação Cuidados Clínicos em Enfermagem e Saúde (PPCCLIS - UECE)
Programa de Pós-graduação em Saúde Coletiva (PPSAC - UECE)

APRESENTAÇÃO

A Enfermagem Escolar é um campo de atuação relativamente novo no Brasil, ainda permeado de incertezas e indefinições, possivelmente pela insuficiência ou mesmo inexistência da referida temática durante a formação do enfermeiro, bem como de regulamentações específicas para o exercício profissional nessa área. Consequentemente, há lacunas de instrumentos e tecnologias de apoio ao desenvolvimento das ações do enfermeiro na escola.

Entretanto, faz-se necessário que os profissionais de enfermagem das instituições educacionais sejam autônomos e construam seu saber/fazer próprio a partir de evidências científicas, ancorado no contexto sociocultural e necessidades dos jovens estudantes para um cuidado integral. Assim, cabe aos profissionais atuantes neste cenário construir essas ferramentas para direcionar suas ações.

Nesse sentido, por tratar-se de uma área de estudo a ser explorada e da necessidade de aprimorar e sistematizar o cuidado realizado pela equipe de enfermagem do Instituto Federal do Ceará (IFCE) com jovens estudantes, desenvolvemos *Pesquisa Convergente Assistencial (PCA)* para construção deste Guia com a participação ativa de enfermeiros atuantes na instituição.

Desse modo, o presente Guia de Enfermagem Escolar foi elaborado com base em estudos de revisão da literatura e pressupostos do *Modelo Teórico de Promoção da Saúde de Nola Pender*. Segue, portanto, os componentes da referida teoria com a finalidade de orientar estratégias e ações no âmbito da prevenção de agravos e na promoção da saúde com os estudantes propiciando-lhes melhorias no desenvolvimento pessoal e estudantil.

Destacamos que o guia foi construído durante a pandemia da COVID-19, na qual se evidenciou a importância da integração dos setores saúde e educação. Enquanto ainda estamos avaliando a extensão dos efeitos nos resultados de saúde e educação do fechamento em massa de escolas, sabemos que resultaram em ansiedade, depressão e sofrimento mental, interrupção da atividade física, aumento da insegurança alimentar e violência entre os estudantes.

Sabe-se que os comportamentos de saúde devem ser estimulados nessa população, visto que eles trazem benefícios imediatos e ao longo da vida. A partir dessa premissa, elaborou-se este guia procurando abordar, inicialmente, o contexto institucional e o retrato da situação de saúde desses jovens estudantes; em seguida, destacam-se estratégias e respectivas ações de promoção da saúde recomendadas no desenvolvimento das práticas de enfermagem escolar.

Descrevemos também a consulta de enfermagem realizada com jovens estudantes no IFCE indicando propostas de promoção da saúde a serem inseridas nos cuidados de enfermagem com jovens estudantes por meio de estratégias para promoção da atividade física, promoção da alimentação saudável, promoção da saúde mental e controle do estresse e estratégias de suporte social para promoção da saúde.

Finalmente, orientamos como e em que momentos o guia pode ser utilizado, destacamos alguns fatores que podem surgir como facilitadores ou barreiras para a utilização do Guia na prática clínica e educativa, bem como apresentamos algumas propostas para difusão e disseminação do conhecimento produzido pelo Guia e trazemos em

apêndice algumas experiências exitosas de ações de promoção da saúde realizadas pelos profissionais de enfermagem do IFCE.

Este Guia destina-se aos profissionais de enfermagem do IFCE que atuam na assistência estudantil. Além disso, também pode ser utilizado por outros profissionais da área de enfermagem e saúde que trabalham na assistência direta aos jovens estudantes em instituições educacionais e contemplam em suas atividades estratégias de promoção da saúde.

Assim, vislumbra-se sua implementação no IFCE, instituição de origem, em que emergiram questionamentos sobre as fragilidades dessas práticas, na integração com a equipe multiprofissional que assiste a população de jovens estudantes. Acredita-se que esse espaço institucional é privilegiado para ações de promoção da saúde com impactos positivos por todo o ciclo de vida dos jovens que forem beneficiados.

Os Autores.

SUMÁRIO

1. ENFERMAGEM ESCOLAR E A PROMOÇÃO DA SAÚDE DE JOVENS ESTUDANTES 08

2. OBJETIVO DO GUIA 10

3. INSTITUTO FEDERAL DO CEARÁ (IFCE), POLÍTICA DE ASSISTÊNCIA ESTUDANTIL E ATUAÇÃO DA ENFERMAGEM 11

4. RETRATO DOS JOVENS ESTUDANTES DO IFCE: aspectos sociodemográficos e comportamentos de saúde 13

5. O MODELO DE PROMOÇÃO DA SAÚDE DE PENDER: conceitos, pressupostos e estratégias orientadoras de comportamentos de saúde 17

6. CONSULTA DE ENFERMAGEM EM PROMOÇÃO DA SAÚDE COM JOVENS ESTUDANTES NO IFCE E COLABORAÇÃO INTERPROFISSIONAL 21

7. PROPOSTAS DE PROMOÇÃO DA SAÚDE PARA AS PRÁTICAS DE ENFERMAGEM COM JOVENS ESTUDANTES 24

- **7.1** Estratégias para promoção da atividade física 24
- **7.2** Estratégias para promoção da alimentação saudável 27
- **7.3** Estratégias para promoção da saúde mental e controle do estresse 31
- **7.4** Estratégias de suporte social para promoção da saúde 36

8. COMO E QUANDO UTILIZAR O GUIA? 39

9. FATORES FACILITADORES E BARREIRAS PARA UTILIZAÇÃO DO GUIA 40

10. DIFUSÃO E DISSEMINAÇÃO DO GUIA DE ENFERMAGEM ESCOLAR 42

REFERÊNCIAS 43

EXPERIÊNCIAS EXITOSAS EM PRÁTICAS DE PROMOÇÃO DA SAÚDE PELA EQUIPE DE ENFERMAGEM DO IFCE 48

CONTATOS DOS SERVIÇOS DE ENFERMAGEM DO IFCE 51

1. ENFERMAGEM ESCOLAR E A PROMOÇÃO DA SAÚDE DE JOVENS ESTUDANTES

A saúde pode ser compreendida como uma produção social, fruto das escolhas pessoais, dos processos coletivos, do contexto social e das condições materiais de vida disponíveis para a sua produção. Dentre os setores que promovem condições para tal, destacam-se a Educação e a Saúde que devem se integrar para esse propósito. A integração entre os setores saúde e educação é necessária para articular saberes e práticas que propiciem melhoria nas condições de vida e na saúde (BRASIL, 2015d).

Nesse sentido, a educação em saúde e a promoção da saúde estão ligadas a uma meta comum – incentivar as pessoas a alcançar um bem-estar, de tal modo que elas possam desfrutar a vida da forma mais saudável possível e evitar doenças. A promoção da saúde também representa aquelas atividades que ajudam as pessoas a desenvolverem recursos que mantêm ou aumentam o bem-estar e a qualidade de suas vidas. É, portanto, uma ação multissetorial que envolve esforços pessoais e interprofissionais presentes em todo o ciclo da vida humana (MURDAUGH; PARSONS; PENDER, 2019).

Experiências nacionais e internacionais mostram que a promoção da saúde requer parcerias entre poder público, setores organizados da sociedade civil, incentivo da iniciativa privada e sensibilização do cidadão, bem como o engajamento dos serviços de saúde, lideranças e organizações comunitárias, essencialmente família e escolas, uma vez que nesses ambientes se iniciam as relações afetivas e socioculturais que repercutem na forma de ser e viver da pessoa (BRASIL, 2015e).

Dentre as ações de promoção da saúde, destacam-se a mudança de comportamento e o empoderamento das pessoas e comunidades. O comportamento de saúde é definido como a atividade realizada pelas pessoas com o objetivo de manter ou melhorar sua saúde, prevenir problemas de saúde ou obter uma imagem corporal positiva (COCKERHAM, 2014). Comportamentos de saúde começam na fase infantojuvenil, repercutindo no crescimento e no desenvolvimento, gerando consequências positivas ou negativas nas fases seguintes (DICK; FERGUSON, 2015; PATTON et al., 2016). É importante estarmos atentos ao fato de que não se constrói uma vida saudável isolado e que as mudanças nos estilos de vida não ocorrem exclusivamente pela vontade e atitude individuais.

Nessa perspectiva, a juventude é compreendida não apenas como uma fase de passagem para a vida adulta. Primeiramente, ela não é uma categoria abstrata, e sim formada por pessoas que têm interesses diferentes segundo as desigualdades e as oportunidades que possuem. Em segundo lugar, todos que a compõem trazem anseios de autonomia, emancipação e uma forma nova de ver e compreender o mundo e nele aportar sua singular contribuição (SOBRINHO et al., 2021).

É fundamental envolver os jovens na melhoria da sua condição de saúde, estimulando a autonomia na escolha de comportamentos saudáveis. Os enfermeiros desempenham um papel importante na promoção da saúde pública em diversos contextos e níveis de atenção, incluindo as instituições educacionais.

A Enfermagem Escolar é considerada uma prática especializada que promove o bem-estar, o sucesso acadêmico, a realização ao longo da vida e a saúde dos alunos. Os enfermeiros escolares desempenham um papel essencial em manter as crianças e jovens saudáveis, seguros e prontos para aprender, sendo, muitas vezes, os únicos profissionais de saúde no ambiente educacional (NASN, 2018).

A literatura internacional mostra que a Enfermagem Escolar está inserida em local estratégico e possui as competências para promover a saúde dos jovens. As atividades de enfermeiros escolares podem ser classificadas em quatro áreas principais: (a) promoção da saúde e prevenção de doenças; (b) triagem e tratamento de questões agudas; (c) manejo das condições crônicas; (d) apoio suporte psicossocial. Ressalta-se ainda que as atividades de enfermagem escolar estão associadas ao aumento da frequência, melhoria da qualidade das escolas e economia de custos (LINEBERRY; ICKES, 2015; BEST; OPPEWAL; TRAVERS, 2018).

Nesse contexto, o compromisso do profissional de enfermagem que atua no contexto escolar vai além do cuidado direto à saúde e deve apoiar os profissionais de educação na identificação e resolução de questões relativas à saúde dos estudantes, por meio da socialização de conhecimentos, fortalecendo a integração de saberes de campos diferentes na perspectiva da interdisciplinaridade. Assim, destacam-se como características importantes da prática de Enfermagem Escolar o exercício da colaboração interprofissional e a coordenação do cuidado de enfermagem, atuando na equipe multiprofissional para mobilizar e desenvolver estratégias de promoção da saúde.

PARA SABER MAIS, CLIQUE NO *LINK* ABAIXO E/OU CONFIRA AS REFERÊNCIAS NO FINAL DO GUIA:

POLÍTICAS DE SAÚDE E EDUCAÇÃO PARA A JUVENTUDE NO BRASIL: INTERSETORIALIDADE E ATUAÇÃO DO ENFERMEIRO

2. OBJETIVO DO GUIA

Orientar as práticas dos profissionais de enfermagem do IFCE por meio de estratégias de promoção da saúde e cuidados com jovens.

3. INSTITUTO FEDERAL DO CEARÁ (IFCE), POLÍTICA DE ASSISTÊNCIA ESTUDANTIL E ATUAÇÃO DA ENFERMAGEM

De acordo com a Lei Nº 11.892, de 29 de dezembro de 2008: “Os Institutos Federais (IFs) são instituições de educação superior, básica e profissional, pluricurriculares e multicampi, especializados na oferta de educação profissional e tecnológica nas diferentes modalidades de ensino, com base na conjugação de conhecimentos técnicos e tecnológicos com as suas práticas pedagógicas” (BRASIL, 2008).

O Instituto Federal de Educação, Ciência e Tecnologia do Ceará (IFCE), com reitoria sediada em Fortaleza, foi criado em dezembro de 2008 mediante a integração do Centro Federal de Educação Tecnológica do Ceará (CEFET) com as Escolas Agrotécnicas Federais de Crato e de Iguatu. O IFCE, vinculado ao Ministério da Educação, é uma autarquia de natureza jurídica, detentora de autonomia administrativa, patrimonial, financeira, didático-pedagógica e disciplinar (IFCE, 2017).

A missão do IFCE é “produzir, disseminar e aplicar os conhecimentos científicos e tecnológicos na busca de participar integralmente da formação do cidadão, tornando-a mais completa, visando sua total inserção social, política, cultural e ética” (IFCE, 2018). Segundo o mesmo documento, o IFCE possui como princípios pedagógicos a pesquisa, o trabalho como princípio educativo, o respeito à diversidade, a interdisciplinaridade e o estímulo à autonomia dos educandos.

Atualmente, o IFCE dispõe de reitoria, 33 *campi* em funcionamento e 1 Polo de Inovação distribuídos conforme Figura 1:

Figura 1 – Distribuição dos *campi* do IFCE

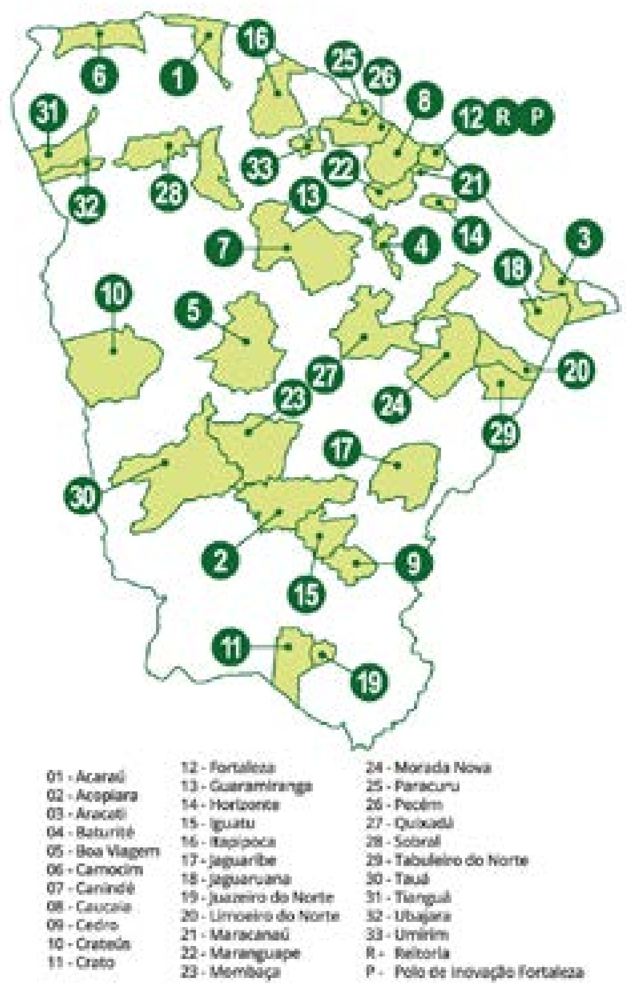

Fonte: IFCE, 2019.

O IFCE dispõe desde 2015 de uma Política de Assistência Estudantil composta por cinco eixos de atuação norteadores: Trabalho, educação e cidadania; Saúde; Alimentação e nutrição; Arte, cultura, desporto e lazer; e os Auxílios em forma de pecúnia. A promoção desses eixos ocorre por meio do desenvolvimento de programas, projetos e ações multiprofissionais e interdisciplinares, direcionados ao desenvolvimento integral do estudante, atendendo às particularidades e aos indicadores sociais dos campi (IFCE, 2015).

O IFCE conta com a Diretoria de Assuntos Estudantis (DAE), criada em 2013, que tem como objetivo planejar, incentivar e apoiar a melhoria do desempenho acadêmico e a educação integral do estudante, reduzindo a evasão e a repetência. A DAE, que fica na Reitoria do IFCE, é responsável pelo gerenciamento sistêmico da Política de Assistência Estudantil, e os *campi* por meio das Coordenadorias de Assuntos Estudantis (CAE) pela sua execução.

As equipes de assistência estudantil são compostas pelos seguintes profissionais, de acordo com a especificidade de cada *campus*: assistentes de alunos, assistentes sociais, educadores físicos, enfermeiros, médicos, nutricionistas, odontólogos, pedagogos, psicólogos, técnicos em assuntos educacionais e técnicos de enfermagem. Inseridas no processo educativo, as atividades das equipes fundamentam-se na articulação com ensino, pesquisa e extensão.

No âmbito do IFCE, a Enfermagem destina-se à promoção da saúde com foco na educação em saúde, bem como a oferecer cuidados de primeiros socorros em situações de urgência e emergência. De acordo com os *Referenciais de Atuação dos Profissionais da Assistência Estudantil do IFCE (2016)*, documento construído coletivamente, destacam-se como principais possibilidades de atuação da Enfermagem:

1. Contribuir para o desenvolvimento integral do(a) discente;
2. Colaborar no mapeamento da realidade socioeconômica, acadêmica e de saúde dos discentes;
3. Apoiar as estratégias de inclusão das pessoas com deficiência;
4. Atuar na prevenção, promoção, tratamento e vigilância à saúde de forma individual e coletiva, colaborando com o processo de ensino-aprendizagem;
5. Realizar ações de prevenção e controle sistemático de situações de saúde e agravos em geral;
6. Desenvolver atividades de educação em saúde para a adoção de hábitos saudáveis, visando à melhoria da qualidade de vida e promoção da saúde comunidade acadêmica;
7. Participar de estratégias de combate à evasão escolar;
8. Participar do planejamento, execução e avaliação da programação das ações anuais de saúde;
9. Participar do processo de seleção de auxílios referente aos aspectos relativos às situações de saúde;
10. Acompanhar os discentes encaminhados para serviços de saúde externos em situações específicas.

PARA SABER MAIS, CLIQUE NO *LINK* ABAIXO E/OU CONFIRA AS REFERÊNCIAS NO FINAL DO GUIA:

[Referenciais de atuação dos profissionais da Assistência Estudantil]

4. RETRATO DOS JOVENS ESTUDANTES DO IFCE: aspectos sociodemográficos e comportamentos de saúde

Segundo dados da Plataforma IFCE em números oriundos do sistema acadêmico do IFCE, atualizados em 20 de julho de 2021, a instituição possui 29.577 matrículas ativas em 357 cursos, incluindo de qualificação profissional, técnico (concomitante, integrado e subsequente), graduação e pós-graduação (IFCE, 2021a).

Com o objetivo de analisar os comportamentos de saúde e fatores associados dos jovens estudantes do IFCE, realizou-se um estudo transversal entre novembro e dezembro de 2020 com 282 estudantes de 18 a 24 anos de sete *campi* do IFCE. Aplicou-se um formulário eletrônico sobre características sociodemográficas, aspectos educacionais e de saúde e o instrumento Adolescent Health Promotion Scale - AHPS (GUEDES; ZUPPA, 2018) composto por seis subescalas relativas à Nutrição, Suporte Social, Responsabilidade pela Saúde, Valorização da Vida, Exercício Físico e Controle do Estresse cujos resultados estão descritos a seguir:

Quanto às características sociodemográficas dos jovens estudantes do IFCE, a média de idade dos participantes da pesquisa foi de 21,3 anos (DP ± 1,8). Catorze estudantes (5%) informaram possuir filho(s) e, destes, 64,3% possuíam apenas um filho. A renda bruta mensal familiar variou de R$ 80,00 a R$ 20.000,00. A mediana da renda bruta mensal familiar foi de R$1.045,00. Além disso, 88 estudantes (31,2%) informaram exercer alguma atividade remunerada e, destes, 50% possuem trabalho formal. Dos alunos, 165 (58,5%) não residem na mesma cidade do *campus* em que estão matriculados. Outras informações estão apresentadas no Infográfico 1.

Infográfico 1 - Características sociodemográficas dos jovens estudantes do IFCE (Fortaleza, Ceará, Brasil, 2021).

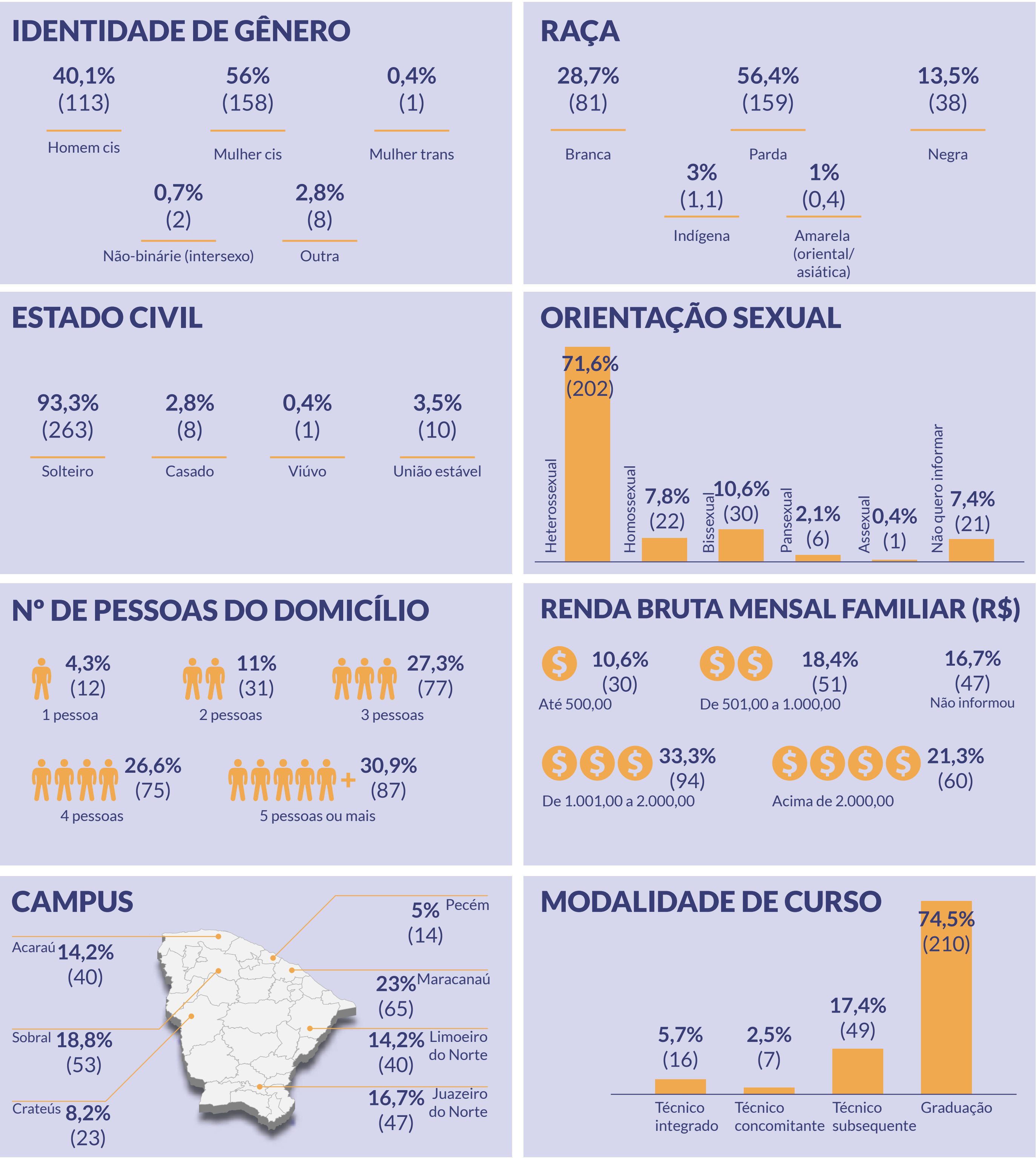

Fonte: dados da pesquisa

No que se refere aos aspectos de saúde e assistência estudantil, 98 estudantes (34,8%) declararam receber alguma bolsa ou auxílio estudantil em forma de pecúnia do IFCE. Dos alunos, 77 (27,3%) informaram possuir algum problema de saúde; destes, 36 (46,7%) mencionaram dois ou mais. Já realizaram atendimento com algum profissional da equipe de assistência estudantil do seu campus 117 estudantes (41,5%); destes, 82 (70,1%) procuraram mais de um profissional. Apenas 61 (21,6%) dos discentes participaram de atividades de educação em saúde com a equipe de enfermagem do seu *campus*. Destaca-se que 69 estudantes (24,5%) se infectaram e/ou moram com alguém que se infectou pelo novo coronavírus. Maiores detalhes estão no Infográfico 2.

Infográfico 2 - Aspectos de saúde e assistência estudantil dos jovens do IFCE (Fortaleza, Ceará, Brasil, 2021).

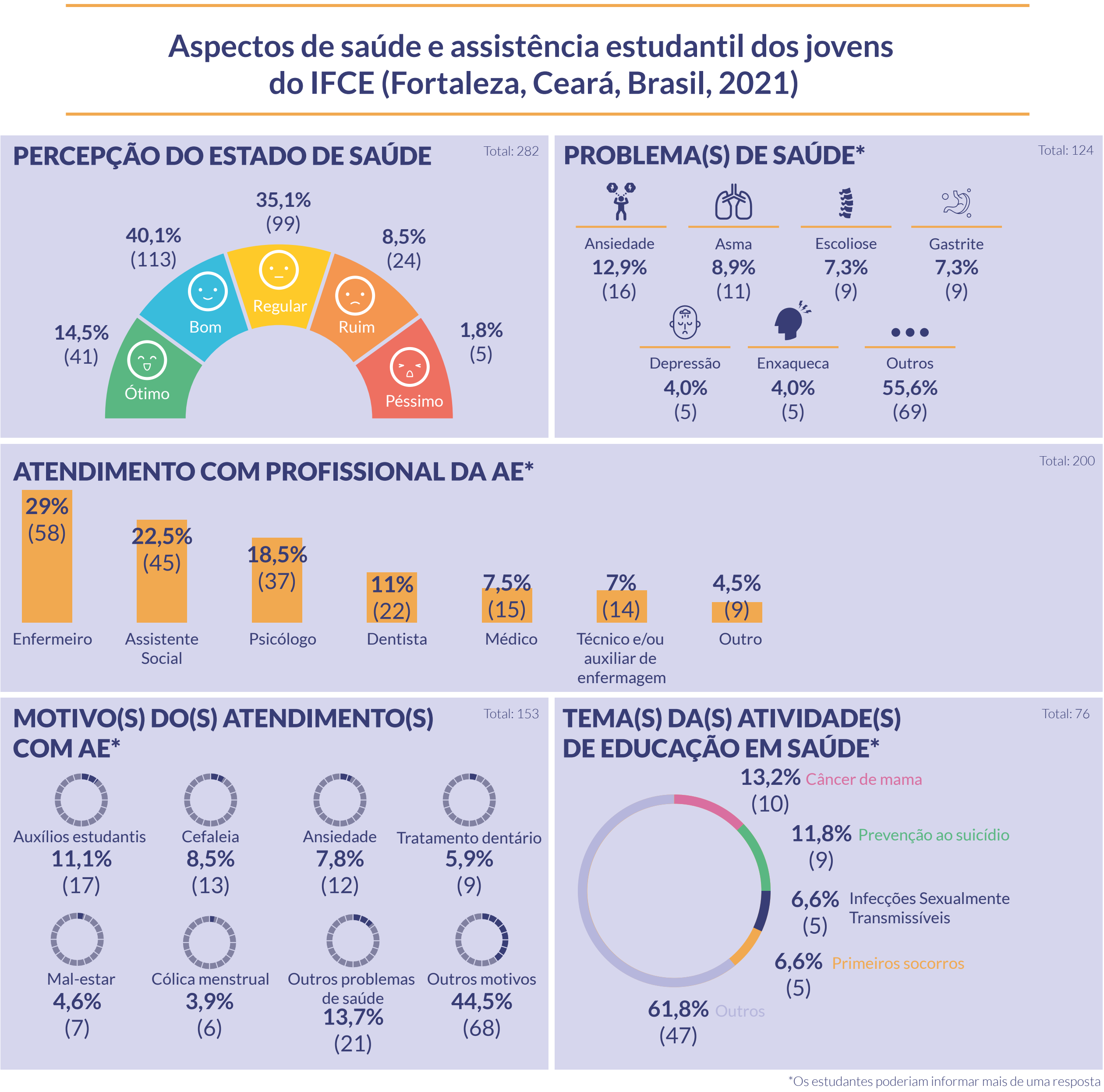

Fonte: dados da pesquisa

A média geral da AHPS foi de 3,29; as subescalas variaram de 2,40 de exercício físico; suporte social e responsabilidade pela saúde 3,05; controle do estresse 3,40; nutrição 3,62; e valorização da vida 3,76. Observaram-se maiores médias na escala geral em estudantes que exercem atividade remunerada, estão matriculados em cursos de graduação e se infectaram pela COVID-19.

Identificamos que os jovens estudantes do IFCE apresentam características sociodemográficas, de saúde e educação semelhantes às de outras pesquisas relacionadas nacionalmente. Com relação aos comportamentos de promoção da saúde, a média da AHPS foi menor que a encontrada em outros estudos realizados anteriormente no Brasil e em outros países, principalmente com adolescentes mais novos e em um cenário pré-pandemia da COVID-19.

Porventura, a maioria dos estudantes não costuma conversar sobre suas preocupações de saúde com profissionais de saúde. Demonstra-se, assim, a necessidade de ampliar o acesso e a divulgação entre os jovens de ações de educação em saúde no IFCE com momentos de escuta e rodas de conversas que propiciem a participação ativa dos jovens nas decisões de sua saúde.

Os comportamentos relacionados ao exercício físico foram os que apresentaram pior desempenho, refletindo o impacto do fechamento das instituições de ensino durante a pandemia da COVID-19 nas práticas de atividade física dos jovens estudantes. Faz-se necessário que os profissionais de saúde e educação reorientam seu cuidado e/ou metodologias de ensino-aprendizagem para incorporar o uso das tecnologias digitais na promoção da saúde de jovens estudantes considerando seus interesses e necessidades.

5. O MODELO DE PROMOÇÃO DA SAÚDE DE PENDER: conceitos, pressupostos e estratégias orientadoras de comportamentos de saúde

O Modelo Teórico de Promoção da Saúde (MPS) foi elaborado por Nola J. Pender em 1982 e revisado por Murdaugh, Parsons e Pender em 2019, propõe uma estrutura para integrar as perspectivas da enfermagem a da ciência comportamental com fatores que predizem comportamentos de saúde. O modelo orienta como explorar processos biopsicossociais que motivam os indivíduos a se engajarem em comportamentos que melhoram a saúde e o bem-estar (MURDAUGH; PARSONS; PENDER, 2019).

O MPS descreve a natureza multidimensional das pessoas que interagem com seus ambientes interpessoais e físicos à medida que buscam a saúde. Ele não inclui o medo ou ameaça como fonte de motivação para o comportamento de saúde.

Os pressupostos do MPS refletem a perspectiva da ciência comportamental e enfatizam o papel ativo do indivíduo na gestão de comportamentos de saúde, alterando o contexto ambiental. O modelo possui uma abordagem ou competência orientada para o foco, aplica-se ao longo do ciclo vital e é útil em diversos contextos. O MPS contribui como uma solução de enfermagem para a política de saúde e para a mudança dos cuidados de saúde fornecendo um meio para compreender como as pessoas podem ser motivadas para alcançar a saúde (MURDAUGH; PARSONS; PENDER, 2019).

O MPS é composto pelos seguintes conceitos:

Características individuais e experiências: incluem fatores pessoais e comportamentos relacionados anterioresque afetam as ações de saúde subsequentes;

Comportamento relacionado anterior: o comportamento anterior igual ou semelhante tem efeitos diretos e indiretos sobre a probabilidade de se engajar em comportamentos promotores de saúde;

Fatores pessoais: incluem fatores biológicos (idade, índice de massa corporal, capacidade aeróbia etc.), fatores psicológicos (autoestima, automotivação, estado de saúde percebido etc.) e fatores socioculturais (gênero, raça, etnia, nível educacional, nível socioeconômico etc.);

Cognições e afetos específicos do comportamento: são considerados de grande importância motivacional e estas variáveis são modificáveis por meio das ações de enfermagem;

Benefícios percebidos da ação: crenças sobre as consequências positivas ou reforçadas da realização de um comportamento. Os benefícios podem ser intrínsecos ou extrínsecos ao indivíduo.

Barreiras percebidas à ação: constituem as crenças sobre a inconveniência, despesa, dificuldade ou natureza demorada de realizar uma determinada ação.

Autoeficácia percebida: é a opinião de um indivíduo sobre a capacidade pessoal de realizar um comportamento específico. É influenciada pelos afetos relacionados à atividade (emoções).

Afeto relacionado à atividade: pode ser positivo ou negativo, é provável que os comportamentos associados ao afeto positivo sejam repetidos, ao passo que aqueles associados ao afeto negativo provavelmente serão evitados.

Influências interpessoais: são pensamentos que envolvem os comportamentos, crenças e atitudes de outras pessoas. As fontes primárias de influência interpessoal nos comportamentos são familiares, pares e prestadores de cuidados de saúde, incluem também normas sociais, expectativas de pessoas importantes, apoio social e modelagem (aprendizagem feita por meio da observação de outras pessoas). A suscetibilidade à influência de outros pode variar no desenvolvimento e ser particularmente evidente na adolescência;

Influências situacionais: percepções e conhecimentos de uma dada situação ou contexto que podem facilitar ou impedir o comportamento. Incluem as percepções das opções disponíveis e características do ambiente em que determinado comportamento é proposto para ocorrer;

Compromisso com um plano de ação: o comprometimento com um plano de ação inicia um comportamento, pois impele os indivíduos à ação, a menos que haja uma demanda conflitante que não possa ser evitada ou uma preferência competitiva que não seja resistida;

Demandas concorrentes imediatas e preferências: as demandas conflitantes são comportamentos alternativos sobre os quais os indivíduos têm um nível relativamente baixo de controle, como responsabilidade no trabalho ou de cuidado familiar. Em contraste, as preferências concorrentes têm propriedades reforçadoras poderosas sobre as quais os indivíduos exercem um nível relativamente alto de controle, dependendo da sua capacidade de auto regulação e controle, por exemplo, escolher um sorvete ou uma fruta para o lanche;

Comportamento promotor de saúde é a ação positiva resultado do MPS, direcionado para obtenção de resultados positivos na saúde. Comportamentos promotores de saúde resultam em melhoria da saúde, capacidade funcional e qualidade de vida em todos os estágios de desenvolvimento. São exemplos de comportamentos promotores de saúde consumir uma alimentação saudável, praticar atividades físicas regularmente, controlar o estresse, repousar adequadamente e construir relações positivas.

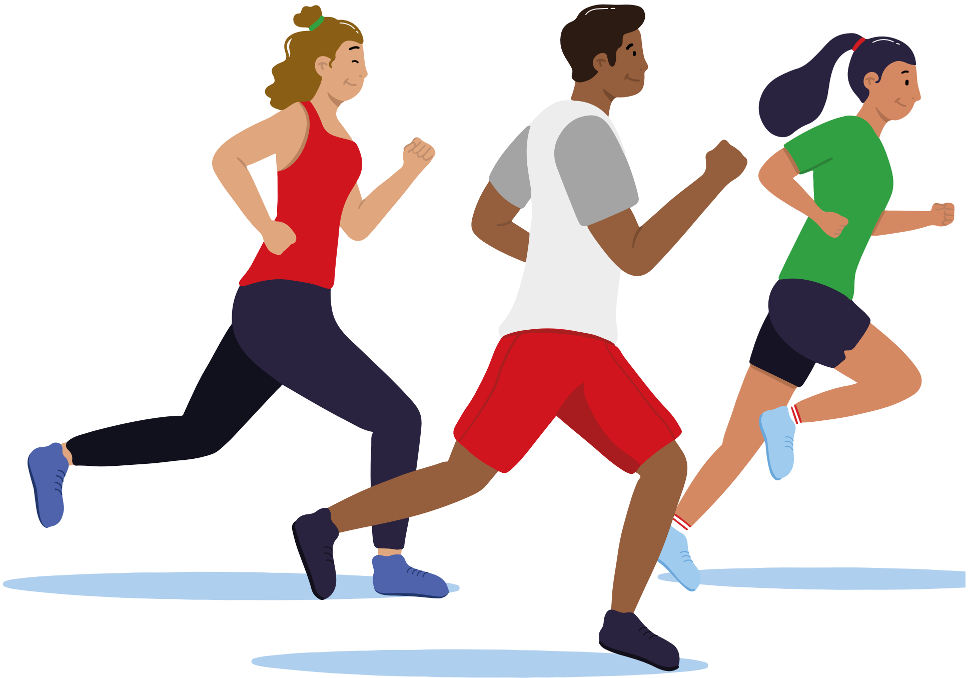

Figura 2 – Diagrama do Modelo de Promoção da Saúde

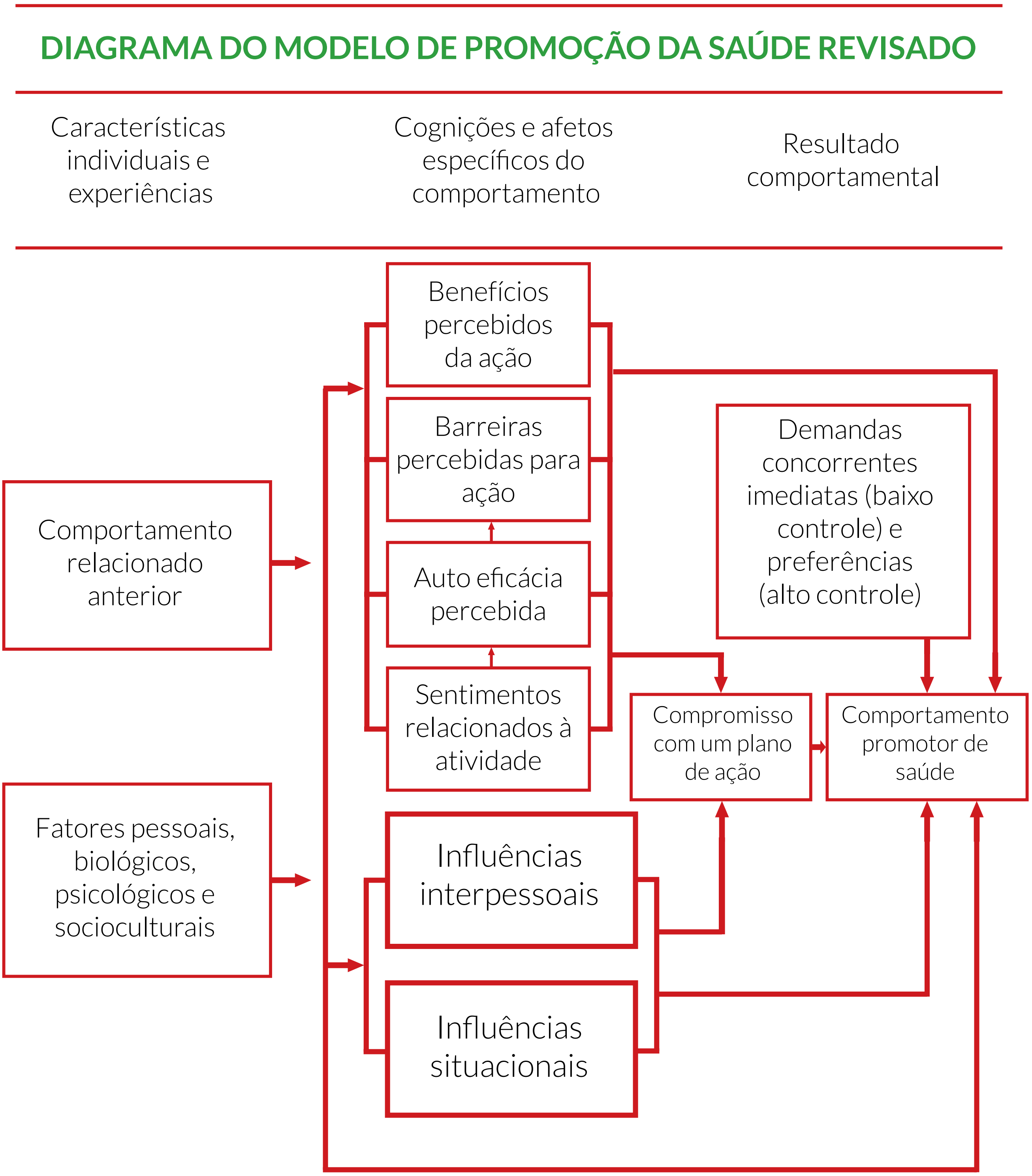

Fonte: MURDAUGH; PARSONS; PENDER, 2019

O MPS também destaca algumas estratégias que podem ser utilizadas para promover mudanças de comportamentos de saúde, como:

- Definir metas para mudança: os indivíduos assumem o compromisso de mudar quando as metas e resultados de comportamentos desejados são definidos e os planos de ação desenvolvidos para atingir as metas.

- Monitorar o progresso em relação aos objetivos para promover a mudança: o automonitoramento ou monitoramento do progresso é uma estratégia comportamental que permite aos indivíduos avaliarem continuamente o progresso em relação ao seu objetivo e fazerem mudanças com base nas informações de monitoramento.

- Promover a autoeficác[illegible] a maneira mais eficiente para alcançar a autoeficácia é por meio do desempenho bem-sucedido (maestria) na realização do comportamento e para isso é necessário praticá-lo com regularidade.

- Gerenciar barreiras para a mudança: a mudança de comportamento é facilitada quando as barreiras à tomada de decisão são minimizadas ou eliminadas de forma consistente.

Esse modelo teórico divide as intervenções de promoção da saúde e prevenção de doenças em quatro eixos principais: **atividade física, nutrição, gerenciamento de estresse e suporte social**. No Brasil, as pesquisas que utilizaram o MPS como referencial teórico trabalham principalmente com adolescentes e jovens estudantes, demonstrando a adequação dessa teoria ao trabalho com o público jovem. No entanto, fazem-se necessários mais estudos, especialmente de intervenções observando os efeitos da aplicação da teoria no contexto da promoção da saúde dessa população.

PARA ACESSAR ALGUMAS PESQUISAS QUE UTILIZARAM O MPS, CLIQUE NOS *LINKS* ABAIXO:

Tecnologia educacional baseada em Nola Pender: promoção da saúde do adolescente

Disponível aqui

Programa de intervenções educativas para prevenção de IST/HIV entre adolescentes e jovens:

Disponível aqui

6. CONSULTA DE ENFERMAGEM EM PROMOÇÃO DA SAÚDE COM JOVENS ESTUDANTES NO IFCE E COLABORAÇÃO INTERPROFISSIONAL

A Consulta de Enfermagem é considerada uma atividade privativa do Enfermeiro, que utiliza componentes do método científico para identificar situações de saúde/doença, prescrever e implementar medidas de Enfermagem que contribuam para a promoção, prevenção, proteção da saúde, recuperação e reabilitação do indivíduo, família e comunidade (BRASIL, 1986).

De acordo com o Conselho Federal de Enfermagem (COFEN), o Processo de Enfermagem (PE) deve ser realizado, de modo deliberado e sistemático, em todos os ambientes, públicos ou privados, em que ocorre o cuidado profissional de Enfermagem, incluindo escolas e outras instituições de ensino. Quando realizado no ambiente escolar, o Processo de Enfermagem corresponde ao usualmente denominado como Consulta de Enfermagem. Ressalta-se que o PE pode ocorrer no contexto da Consulta de Enfermagem e nos diversos encontros entre o enfermeiro e a população a ser cuidada (COFEN, 2009).

No IFCE, realizam-se Consultas de Enfermagem para avaliação/acompanhamento da saúde dos discentes, prioritariamente para os ingressantes e/ou beneficiados por auxílios estudantis. Os atendimentos são agendados no contraturno ou no horário de aula de acordo com a disponibilidade dos estudantes. Nas avaliações de saúde, ocorre a identificação de problemas de saúde, promoção do autocuidado, além do encaminhamento do estudante para a rede de saúde, se necessário.

Na Consulta de Enfermagem, usualmente, coleta-se o histórico de enfermagem, faz-se exame físico geral, bem como avaliação avaliação antropométrica, verificação da glicemia capilar e dos sinais vitais, avaliação da acuidade visual por meio da escala de Snellen, verificação da situação vacinal, identificação e acompanhamento de estudantes com deficiência, necessidades específicas e/ou com agravos/doenças crônicas.

Destaca-se que as Consultas de Enfermagem precisam estimular o protagonismo dos estudantes, de modo a obter um envolvimento ativo e escolhas conscientes das suas atitudes e comportamentos de saúde. Observa-se que as avaliações são mais bem-sucedidas quando se convertem em processos de educação em saúde para que haja continuidade do cuidado. Assim, não basta avaliar e identificar problemas, o compromisso e a autonomia dos jovens para o seu autocuidado são importantes. No atendimento, precisa-se considerar a etapa de crescimento e desenvolvimento dos jovens e aspectos relativos à sua saúde mental. Além disso, o envolvimento da família e responsáveis na consulta e demais ações aumenta o alcance e efetividade do cuidado.

A Consulta de Enfermagem tem sido uma atividade importante desenvolvida no cuidado à saúde de jovens no contexto do IFCE, no entanto a promoção da saúde vai além da triagem e inclui extensos esforços para promover comportamentos de saúde desde uma idade precoce. Nesse sentido, recomenda-se integrar alguns pressupostos do MPS à Consulta de Enfermagem, buscando identificar e intervir, quando possível, nos fatores pessoais, benefícios e barreiras percebidas, influências interpessoais e situacionais sobre os comportamentos de saúde dos jovens estudantes.

Faz-se necessário considerar no planejamento das ações as experiências individuais, os sentimentos e conhecimentos acerca do comportamento de saúde específico, além de determinar a autoeficácia percebida e a autopercepção de saúde. O MPS pode auxiliar o enfermeiro a compreender os fatores relacionados com o processo biopsicossocial que influenciam o comportamento do sujeito a ter um estilo de vida saudável a fim de que seja possível propor ferramentas necessárias para a mudança nesse comportamento (BITTENCOURT; MARQUES; BARROSO, 2018).

Seguindo uma tendência mundial, as práticas de enfermagem escolar envolvem trabalho e colaboração interprofissional, ou seja, profissionais que querem trabalhar juntos para oferecer uma melhor atenção à saúde, o que pode se dar na própria equipe de assistência estudantil e em rede intersetorial com a comunidade (PEDUZZI; AGRELI, 2018). O trabalho interprofissional denota um grau maior de interação e de integração da equipe por meio de comunicação efetiva e compartilhamento de responsabilidades e práticas para atender às necessidades dos estudantes.

Apresentamos em Apêndice algumas experiências exitosas de cuidados de enfermagem e atividades educativas desenvolvidas pela Equipe de Enfermagem do IFCE com foco na promoção da saúde dos jovens estudantes.

PARA SABER MAIS, CLIQUE NO *LINK* ABAIXO E/OU CONFIRA AS REFERÊNCIAS NO FINAL DO GUIA:

[Processo de Enfermagem: Guia para a Prática]

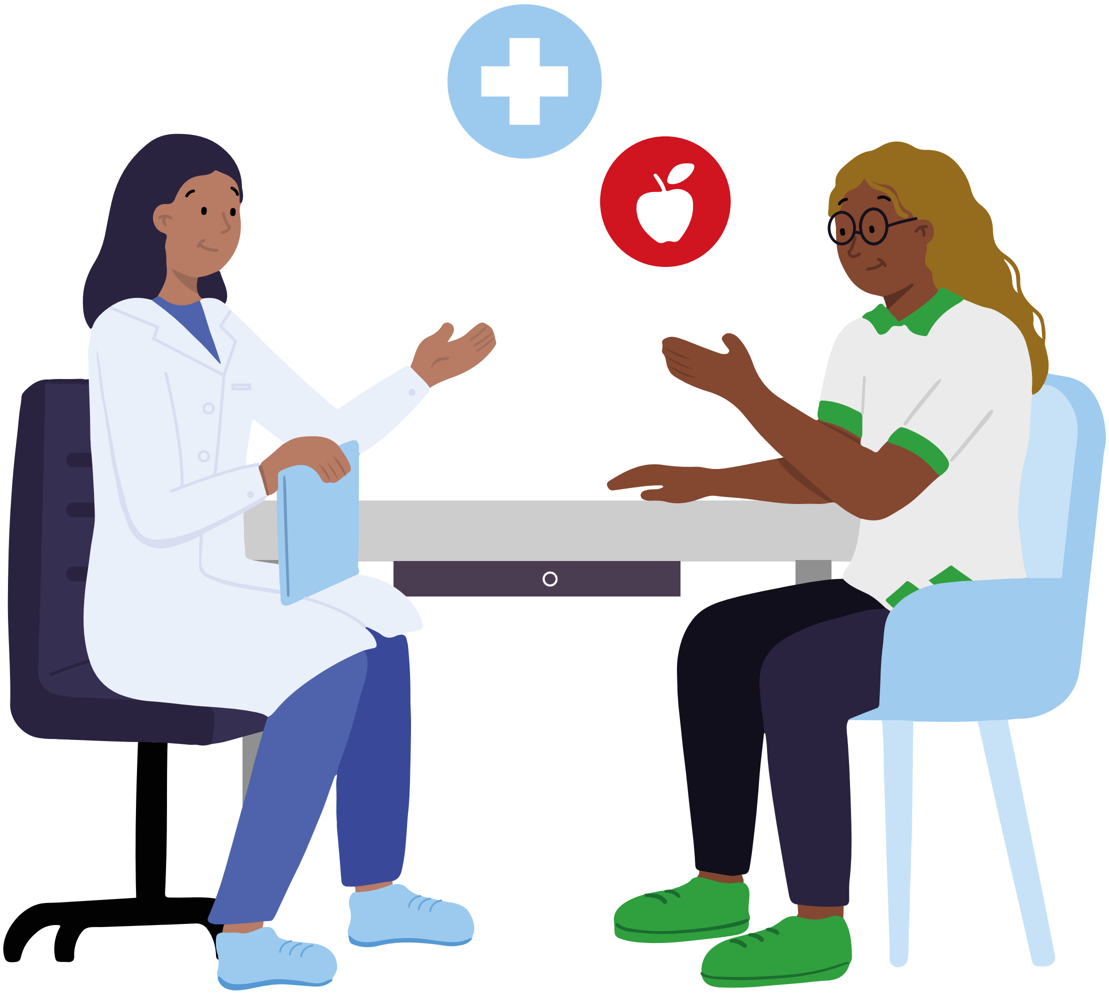

7. PROPOSTAS DE PROMOÇÃO DA SAÚDE PARA AS PRÁTICAS DE ENFERMAGEM COM JOVENS ESTUDANTES

O MPS classifica as intervenções de promoção da saúde e prevenção de doenças em quatro eixos principais: atividade física, nutrição, gerenciamento do estresse e suporte social. Assim, no Guia, optou-se por utilizar a mesma classificação para organizar as estratégias e ações propostas. No entanto, há outras temáticas relacionadas à promoção da saúde que podem ser desenvolvidas pelos enfermeiros no contexto escolar, como a saúde sexual e reprodutiva, uso de substâncias, doenças crônicas não transmissíveis, dentre outras, por serem necessárias a essa população.

7.1 Estratégias para promoção da atividade física

A atividade física é definida como um comportamento que envolve movimentos corporais voluntários gerados pelos músculos esqueléticos que demandam gasto de energia acima do nível de repouso, promovendo interações sociais e com o ambiente. A atividade física é importante para o pleno desenvolvimento humano e deve ser praticada em todas as fases da vida e em diversos momentos, como ao se deslocar de um lugar para outro, durante o trabalho ou estudo, ao realizar tarefas domésticas ou durante o tempo livre. A prática regular de atividade física é essencial para uma vida saudável, energética e produtiva (BRASIL, 2021a).

A atividade física está relacionada à saúde, no seu sentido mais amplo, incluindo aspectos individuais e coletivos, como também ao lazer, à qualidade de vida e à educação, devendo ser encarada como um direito que impacta no desenvolvimento humano. A adesão a um estilo de vida fisicamente ativo não depende somente de uma decisão individual, mas de oportunidades e da inter-relação de diversos fatores individuais, coletivos, ambientais, sociais, culturais, econômicos e políticos. Dessa forma, estratégias para superar as barreiras e promover a prática de atividade física são fundamentais, incluindo as ações vinculadas ao contexto escolar e comunitário (BRASIL, 2015d).

Os exercícios físicos também são exemplos de atividades físicas, mas se diferenciam por serem atividades planejadas, estruturadas e repetitivas com o objetivo de melhorar ou manter as capacidades físicas e o peso adequado, além de serem prescritos por profissionais de educação física (BRASIL, 2021a). Todo exercício físico é uma atividade física, mas nem toda atividade física é um exercício físico!

Quanto mais cedo a atividade física é incentivada e se torna um hábito de vida, maiores os benefícios para a saúde. Alguns desses benefícios são: o controle do peso; a diminuição da chance de desenvolvimento de alguns tipos de cânceres e de doenças crônicas, como a diabetes, hipertensão e cardiopatias; a melhora da disposição e a promoção da interação social (BRASIL, 2015d). Para crianças e adolescentes, ela proporciona muitos benefícios à saúde, seja imediato ou em longo prazo, como melhoria da aptidão cardiovascular e nos processos cognitivos, aumento da massa óssea e do bem-estar mental (MURDAUGH; PARSONS; PENDER, 2019).

Segundo o Guia de Atividade Física para a População Brasileira, as crianças e jovens de 6 a 17 anos devem praticar 60 minutos ou mais de atividade física por dia, incluindo, em pelo menos 3 dias na semana, atividades de fortalecimento dos músculos e ossos, tais como musculação. Para adultos de 18 a 59 anos, a recomendação é praticar, pelo menos, 150 minutos por semana de atividades físicas moderadas ou 75 minutos de atividades vigorosas por semana, bem como incluir, em pelo menos 2 dias na semana,

atividades de fortalecimento dos músculos e ossos (BRASIL, 2021a).

A educação física pode contribuir de forma significativa para a saúde e para o desenvolvimento pessoal dos estudantes. Participar das aulas de educação física vai além da prática de atividade física e do desenvolvimento de habilidades motoras, contribuindo para uma vida ativa e saudável. A educação física de qualidade deve ser obrigatória, preferencialmente ministrada por um professor de educação física, ao longo de todos os anos da Educação Básica. Devem ser oferecidas, pelo menos, três aulas de educação física de 50 minutos cada, por semana (BRASIL, 2021a).

PARA SABER MAIS, CLIQUE NOS *LINKS* ABAIXO E/OU CONFIRA AS REFERÊNCIAS NO FINAL DO GUIA:

GUIA DE ATIVIDADE FÍSICA PARA A POPULAÇÃO BRASILEIRA

Ministério da Saúde lança Guia de Atividade Física para a População Brasileira

Propostas de ações:

- Obtenção de dados sobre o comportamento de atividade/exercício físico dos estudantes;
- Realização de aconselhamento sobre atividade física para os estudantes em colaboração com professores de educação física, destacando os benefícios da atividade física;
- Encaminhamento para o professor de educação física e/ou outros profissionais/serviços da Rede de Atenção à Saúde (RAS) quando se tratar de uma situação que exija orientação/atendimento especializado, como no caso de estudantes com deficiência;
- Incentivo à participação dos estudantes nas disciplinas e aulas de educação física;
- Apoio à realização de eventos esportivos internos e externos como jogos, campeonatos, corridas, passeios ciclísticos e trilhas em parceria com os professores de educação física e/ou com a comunidade externa;
- Criação de ambientes saudáveis no contexto escolar que estimulem/favoreçam a prática de atividade física (oportunidades ativas);
- Estímulo à cultura corporal do movimento no contexto escolar por meio de práticas de lutas, esportes, jogos, brincadeiras, ginásticas e práticas rítmicas;
- Sensibilização da comunidade acadêmica do IFCE sobre a importância da promoção da atividade física e sua integração no currículo escolar;
- Postagens nas redes sociais com orientações sobre atividade física;
- Elaboração de pôsteres temáticos e boletins de notícias sobre a prática de atividade física;
- Estruturação de projetos de intervenção de base escolar para a promoção de um estilo de vida saudável;
- Realização de debates e discussões sobre

políticas públicas brasileiras voltadas para à promoção da saúde;

- Auxílio na realização de intervalos ativos no IFCE com a disponibilização de materiais e equipamentos esportivos, como mesa de pingue-pongue, acesso a pátios, quadras ou ginásios;
- Apoio à liberação do acesso às quadras, piscinas, academias e demais espaços da instituição para prática de atividade física fora do horário de aula e nos finais de semana (analisar a necessidade de supervisão);
- Estímulo ao uso preferencial de escadas ao invés do elevador;
- Incentivo à implementação de, no mínimo, três aulas de educação física semanais no ensino básico;
- Apoio às representações estudantis para que protagonizem as decisões e ações voltadas à promoção da atividade física;
- Criação e/ou divulgação de projetos relacionados a esporte, dança e lazer com jovens;
- Realização de parcerias com as equipes de atenção primária à saúde, Programas Academia da Saúde, Saúde na Escola, Segundo Tempo e o Esporte e Lazer na Cidade;
- Identificar e apoiar movimentos populares de dança, lutas, entre outros, na comunidade, que também são possibilidades de prática de atividade física no contexto local;
- Identificar espaços comunitários acessíveis para a prática de atividade física e mobilizar os estudantes para sua utilização e manutenção;
- Desenvolvimento de espaços de troca de experiências entre os profissionais e os estudantes (fóruns, mostras, seminários, rodas de conversa, entre outros) para compartilhar vivências e resultados da atividade física;
- Promoção do uso adequado de dispositivos eletrônicos, por exemplo, televisão, internet e jogos;
- Apoio à construção e/ou qualificação das instalações esportivas do IFCE;
- Utilização da técnica de Entrevista Motivacional[1] com foco na atividade física.

[1] A Entrevista Motivacional é um estilo de conversa colaborativa que almeja o fortalecimento da motivação da pessoa e seu comprometimento com a mudança de comportamento de saúde. Para saber mais, acesse o artigo disponível em: http://pepsic.bvsalud.org/pdf/bapp/v34n87/a11.pdf

Observações:

- Considerar como as questões relacionadas ao gênero influenciam as práticas de atividade física. Seguindo uma tendência mundial, no Brasil, os rapazes são mais ativos fisicamente que as moças. Em geral, eles participam mais de atividades vigorosas, por exemplo, jogar bola e correr; já elas preferem atividades de baixa intensidade, por exemplo, caminhar e dançar. A instituição de ensino deve problematizar e superar essa dicotomia, ampliando as possibilidades de práticas corporais para ambos os gêneros, pode-se considerar a possibilidade de atividades e instalações específicas por gênero;

- Incluir os estudantes com deficiência e/ou menos habilidades motoras ou cognitivas no planejamento das ações de promoção da atividade física, realizando as adaptações necessárias e englobando os jovens de forma integral;

- Considerar a faixa etária e etapa do desenvolvimento dos estudantes, a pertinência das práticas propostas para o universo dos jovens daquela instituição de ensino e a variedade das práticas corporais;

- É fato a construção social dos comportamentos de saúde, que podem assumir vários formatos e significados internos e externos aos sujeitos. Lidar com essa possibilidade é assumir que aumentar o nível de atividade física de jovens depende de elementos objetivos que definem as escolhas, como segurança e iluminação pública, espaços adequados, apoio social e, sobretudo, conhecimento sobre o fenômeno para produzir adesão, significado da prática para o sujeito, seja momentâneo ou duradouro;

- O suporte social – família, amigos e demais pessoas de referência – contribui significativamente na adesão a hábitos relacionados à atividade física;

- É fundamental que os estudantes compreendam os conceitos básicos relacionados com a saúde e atividade física, que sintam prazer na prática de atividades físicas e que desenvolvam certo grau de habilidade motora, o que lhes dará a percepção de competência e motivação para essa prática (autoeficácia).

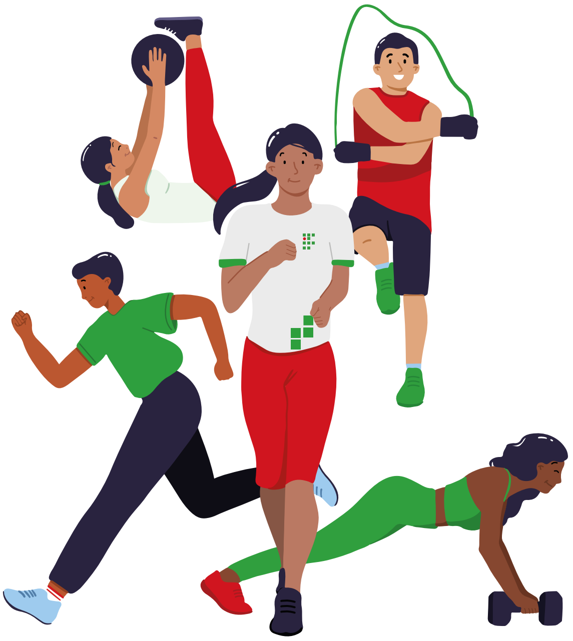

7.2 Estratégias para promoção da alimentação saudável

Alimentação e nutrição constituem requisitos básicos para a promoção e proteção da saúde, pois possibilitam a realização do pleno potencial de crescimento e desenvolvimento humano com qualidade de vida e cidadania (BRASIL, 2015e). A boa nutrição é um dos principais determinantes da saúde e contribui significativamente para a redução das doenças crônicas. A nutrição saudável para crianças e adolescentes é fundamental, uma vez que os padrões alimentares que se desenvolvem durante esses anos tendem a persistir ao longo da vida.

A alimentação adequada e saudável é um direito humano básico que envolve a garantia ao acesso permanente e regular, de forma socialmente justa, a uma prática alimentar adequada aos aspectos biológicos e sociais do indivíduo e que deve estar em acordo com as necessidades alimentares especiais; ser referenciada pela cultura alimentar e pelas dimensões de gênero, raça e etnia; acessível do ponto de vista físico e financeiro; harmônica em quantidade e qualidade, atendendo aos princípios da variedade, equilíbrio, moderação e prazer; e baseada em práticas produtivas adequadas e sustentáveis (BRASIL, 2014).

Pode-se entender a promoção da alimentação saudável como um conjunto de estratégias que proporcionam às pessoas e coletividades a realização de práticas alimentares apropriadas aos seus aspectos biológicos e socioculturais, bem como ao uso sustentável do meio ambiente, tendo como objetivo a melhoria da qualidade de vida da população por meio de ações intersetoriais voltadas ao coletivo, a indivíduos e aos ambientes (físico, social, político, econômico e cultural), de caráter amplo e que possam atender às especificidades de cada fase do curso da vida (BRASIL, 2015a).

Os estudantes do IFCE são, em grande parte, adolescentes e jovens. Essa fase é caracterizada por diversas alterações aceleradas de natureza fisiológica e hormonal que afetam as necessidades nutricionais, tais como crescimento rápido, ganho de massa muscular e óssea. Portanto, nessa fase, a alimentação deve sustentar o crescimento e promover a saúde.

O cuidado com a maneira que temas como sobrepeso e obesidade são abordados pode ser determinante para a relação que os estudantes irão estabelecer com a comida e a prática de refeições. Estas são questões que não podem se configurar como elementos estigmatizadores de padrões físicos, já que podem desencadear outros problemas sérios referentes à imagem corporal que é construída.

Em nenhuma época o corpo magro e esbelto esteve tão em evidência como nos tempos atuais, podendo desencadear graves consequências, como os transtornos alimentares. Trata-se de desordens complexas, causadas e mantidas por diversos fatores sociais, psicológicos e biológicos. Afetam mais a população feminina, sendo que em mulheres adultas, revela-se no desejo generalizado de um corpo mais magro, e em crianças e adolescentes estão associados com baixa autoestima, solidão e ineficácia social. Os mais comuns são a anorexia nervosa, a bulimia nervosa e o transtorno da compulsão alimentar periódica. Os rapazes também são afetados, construindo uma autoimagem deturpada, considerando-se em geral mais fracos e magros. Constituem desse modo outro grupo de risco para os Transtornos Alimentares, mas nesse caso, caracterizado pelo desejo de um corpo mais musculoso, mais forte, conhecido como vigorexia (BRASIL, 2015e).

Nesse contexto, a alimentação escolar balanceada e saudável e a educação alimentar e nutricional podem exercer influência positiva no rendimento escolar, uma vez que aumenta a capacidade de concentração e desenvolvimento cognitivo dos estudantes.

O Programa Nacional de Alimentação Escolar (PNAE) é uma política pública que objetiva contribuir para o crescimento, o desenvolvimento, a aprendizagem e a formação de práticas alimentares saudáveis dos estudantes por meio da oferta de refeições que contemplem as suas necessidades nutricionais durante o período que eles estão na escola e de ações de educação alimentar e nutricional (BRASIL, 2015e).

A Educação Alimentar e Nutricional é definida no contexto da realização do direito humano à alimentação adequada e da garantia da Segurança Alimentar e Nutricional como “um campo de conhecimento e prática contínua e permanente, intersetorial e multiprofissional, que visa promover a prática autônoma e voluntária de hábitos alimentares saudáveis” (BRASIL, 2015e).

PARA SABER MAIS, ACESSE OS *LINKS* ABAIXO E/OU CONFIRA AS REFERÊNCIAS NO FINAL DO GUIA:

Guia Alimentar para a População Brasileira

Você conhece o Guia Alimentar para a População Brasileira?

Princípios do Guia Alimenta para população brasileira

Propostas de ações:

- Realização de debates, oficinas e rodas de conversa sobre a importância de consumir preferencialmente alimentos in natura ou minimamente processados, em grande variedade e predominantemente de origem vegetal;
- Oferta de minicursos sobre alimentação e estilo de vida saudável;
- Construção de materiais educativos (cartilhas, guias, manuais, que possam colaborar nas ações com os jovens estudantes);
- Desenvolvimento e/ou utilização de tecnologias da informação e comunicação, como jogos virtuais, aplicativos, *websites* de apoio às atividades educativas;
- Avaliação antropométrica (peso, altura, índice de massa corporal), glicemia de jejum e do estado nutricional dos estudantes pelo menos uma vez por ano;
- Promover o aumento da ingestão de água, estimulando o consumo de, no mínimo, 2 litros de água diariamente para adultos;
- Orientação/aconselhamento sobre nutrição e padrão de ingestão de alimentos de modo coletivo e individual;
- Encaminhamento para o(a) nutricionista e/ou outros profissionais/serviços da RAS quando se tratar de uma situação que exija orientação/atendimento especializado, como intolerância/alergia alimentar;

- Encaminhamento e apoio para tratamento de transtornos alimentares, por exemplo, anorexia, bulimia e vigorexia;
- Estímulo à oferta de alimentação saudável e saborosa aos estudantes nas cantinas e refeitórios da instituição, como frutas frescas ou secas ou outros alimentos *in natura* ou minimamente processados ou "refeição feita na hora", e restrição à oferta de alimentos ricos em açúcar, sal e gorduras;
- Promoção da redução do consumo de açúcar e bebidas adoçadas com açúcar;
- Promoção da participação ativa dos estudantes na elaboração/adaptação e escolha do cardápio de lanches e/ou refeições oferecidas pelos campi;
- Utilização do *Guia Alimentar para a População Brasileira* nas atividades educativas comparando as práticas alimentares realizadas pelos estudantes com as recomendadas pelo guia para posterior orientação sobre os grupos alimentares e o valor nutricional dos alimentos;
- Estabelecimento do "Dia da fruta" para oferta exclusiva desse item no lanche aos alunos;
- Estímulo para que os estudantes realizem refeições acompanhados por familiares e amigos sempre que possível;
- Promoção da inclusão do tema alimentação e saúde, habilidades culinárias e tradições culinárias locais no currículo escolar e em reuniões de pais e professores;
- Colaboração no acompanhamento da execução do Programa Nacional de Alimentação Escolar (PNAE) e sua integração com a agricultura familiar na instituição;
- Organização/participação em hortas comunitárias ou escolares para produção de alimentos orgânicos ou com base agroecológica, propiciando aos estudantes a construção de conhecimentos e habilidades que lhes permitem produzir, descobrir, selecionar e consumir os alimentos de forma adequada, saudável, segura e lúdica;
- Estímulo à eliminação de propagandas de alimentos processados ou ultraprocessados na instituição buscando diminuir o efeito da publicidade no comportamento alimentar;
- Atuação com o profissional nutricionista/serviço de alimentação e nutrição da instituição visando oferecer locais limpos, confortáveis e tranquilos para que os estudantes façam suas refeições/lanches;
- Obtenção de dados sobre ingestão de alimentos, apetite e preferências alimentares dos estudantes;
- Utilização da técnica de entrevista motivacional com foco na alimentação saudável;

Observações:

- As ações devem priorizar três áreas estratégicas: os conhecimentos, as atitudes e os comportamentos alimentares;

- Antes de planejar e/ou implementar qualquer estratégia de promoção da alimentação saudável com jovens estudantes, é importante compreender a concepção dos jovens sobre alimentação saudável, identificar/analisar o perfil nutricional desses jovens, entender como a aquisição e preparação de alimentos são realizadas por eles e suas famílias, bem como conhecer as barreiras e benefícios percebidos para adotar uma alimentação saudável.

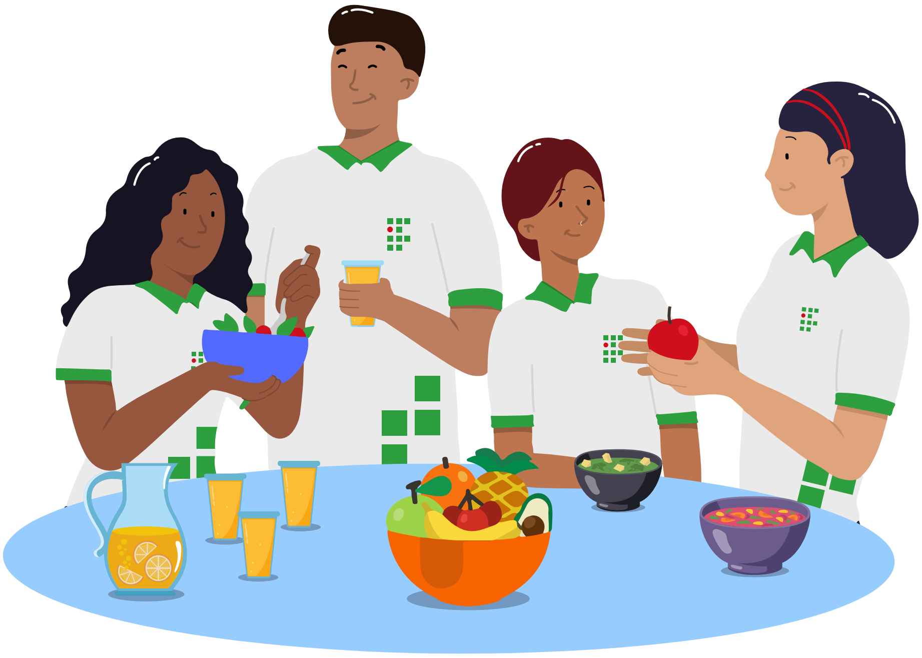

7.3 Estratégias para promoção da saúde mental e controle do estresse

Promover o bem-estar psicológico e proteger os jovens de experiências adversas e fatores de risco que possam afetar seu potencial de prosperar são indispensáveis e as intervenções para promover a saúde mental dos jovens visam fortalecer os fatores de proteção e melhorar as alternativas aos comportamentos de risco.

A promoção da saúde mental e do bem-estar ajuda esse grupo a construir resiliência para que possam lidar bem com situações difíceis ou adversidades. Programas de promoção da saúde mental para jovens exigem uma abordagem multinível com plataformas de distribuição variadas, por exemplo, mídias digitais, ambientes de saúde ou assistência social, escolas ou comunidade (OPAS, 2021).

Dentre as principais características do estado de saúde mental dos jovens

nas Américas, destaca-se o elevado número de mortes por suicídio, além de níveis significativos de problemas de saúde mental, introdução precoce e níveis consideráveis de uso atual e uso excessivo de álcool entre os jovens da região. Além disso, vários estudos demonstram a deterioração da saúde mental em crianças e jovens durante a pandemia da COVID-19, incluindo níveis elevados de solidão, medo, estresse, ansiedade e depressão, causados ou exacerbados por medidas de distanciamento social. Para lidar com os fatores de estresse provocados pela pandemia, os jovens podem recorrer a mecanismos nocivos, como o uso de substâncias (OPAS, 2021b).

Estudo sobre a carga dos transtornos mentais no Brasil identificou que durante a juventude e idade adulta os distúrbios mais comuns são ansiedade, depressão, autoagressão e transtorno somático, seguidos pelos transtornos por uso de substâncias, dores de cabeça e transtornos mentais graves, como esquizofrenia e transtorno bipolar (OPAS, 2018).

O controle e a redução do estresse são aspectos importantes na promoção da saúde. Estresse é a maneira como o organismo responde a qualquer estímulo – bom, ruim, real ou imaginário – que altere seu estado de equilíbrio. Para se entender o que é estresse, é preciso que se entenda o que é homeostase – o estado de equilíbrio dos vários sistemas do organismo entre si e do organismo como um todo com o meio ambiente (NAHAS, 2017).

Estresse é, portanto, um aspecto natural da própria vida. Constantemente, avaliamos e lidamos com situações mutáveis, a meta desejada é a adaptação ou ajuste à mudança, de tal forma que a pessoa fique novamente em equilíbrio e tenha energia e capacidade de satisfazer as novas demandas. Esse é o processo de enfrentamento do estresse que apresenta componentes fisiológicos e psicológicos.

O estresse também é entendido como uma excessiva carga emocional que neutraliza a capacidade do sujeito de resolver problemas, decorre de causas internas (relacionadas ao indivíduo) e externas (ambientais). A vida não é linear, mas sim transitória e marcada por crises que geram desequilíbrios. Crises fazem parte do desenvolvimento humano, são bastante comuns durante a juventude e favorecem a utilização de recursos adaptativos.

De acordo com Nahas (2017), os principais sintomas do estresse são:

- Cansaço constante;
- Insônia;
- Sensação de esgotamento (incapacidade);
- Ansiedade;
- Falta de concentração;
- Falta de motivação;
- Perda de memória;
- Irritabilidade excessiva;
- Mau humor;
- Dores musculares e de cabeça;
- Falta ou excesso de apetite.

As situações de estresse, quando fora de controle, podem interferir em nossas atividades diárias, resultando em perda de produtividade e afetando nossos relacionamentos. O aumento contínuo de problemas de saúde relacionados ao estresse, como ansiedade, depressão, automutilação e suicídio, principalmente entre adolescentes e jovens, destaca a importância de os enfermeiros e demais profissionais de saúde

promoverem a saúde mental, aumentando a resistência a situações indutoras de estresse em indivíduos e famílias.

Entre os fatores que contribuem para o estresse durante esse momento da vida, estão o desejo de uma maior autonomia, pressão para agir conforme os pares, exploração da identidade sexual e maior acesso e uso de tecnologias. O contexto escolar pode atuar como fator estressor ou moderador do estresse dependendo das relações que são estabelecidas nesse ambiente, da estrutura física e organizacional da instituição de ensino.

Apesar de não se poder evitar todas as situações de estresse, existem várias maneiras de se responder a elas, reduzindo ou modificando seus efeitos. As características do indivíduo e dos agentes estressantes determinarão as formas mais apropriadas para lidar com o estresse. Destaca-se a necessidade de incentivar as pessoas a gerenciar adequadamente o estresse e reduzir as pressões que são desencadeadoras de transtornos relacionados ao estresse.

As abordagens para gerenciamento do estresse focam na redução da frequência de situações indutoras de estresse por meio da mudança de ambiente, evitar mudanças excessivas, controle e gerenciamento do tempo, bem como aumentar a resistência ao estresse por meio da prática de exercícios físicos, melhorando, dessa forma, a autoestima e autoeficácia, aumentando a assertividade, estabelecendo metas realistas e construindo recursos de enfrentamento ao estresse (MURDAUGH; PARSONS; PENDER, 2019).

A promoção e a realização de rastreios precoces e intervenções específicas de acordo com o estágio de desenvolvimento são necessárias porque crianças, adolescentes, adultos jovens e adultos mais velhos desenvolvem e usam diferentes estratégias de enfrentamento. A consciência dessas diferenças garante que o enfermeiro intervenha no momento adequado e com a estratégia adequada para alcançar a redução do estresse.

Dentre as estratégias que podem ser utilizadas para o controle do estresse, destacam-se as Práticas Integrativas e Complementares em Saúde (PICS), definidas como recursos terapêuticos que buscam a prevenção de doenças e a recuperação da saúde, com ênfase na escuta acolhedora, no desenvolvimento do vínculo terapêutico e na integração do ser humano com o meio ambiente e a sociedade (BRASIL, 2015c).

As práticas corporais relacionadas às PICS têm potencialidades para a saúde, expressas em sensações de relaxamento, alívio de dores, aumento do bem-estar, diminuição da ansiedade e estresse, melhora do sono, impactos sobre sintomas de doenças, além de contribuições para os processos de autocuidado e ressignificação das formas de conceber e lidar com os processos saúde-doença, levando em consideração dimensões materiais e imateriais do corpo (ANTUNES et al., 2018).

Atualmente, 29 práticas foram institucionalizadas por meio da Política Nacional de Práticas Integrativas e Complementares no SUS - PNPIC (BRASIL, 2015c). Alguns exemplos de PICS são a massoterapia, meditação, musicoterapia, tai chi chuan, terapia comunitária integrativa e yoga segundo o Glossário Temático (BRASIL, 2018). Algumas PICS já são desenvolvidas em alguns campi pelos enfermeiros do IFCE e estão descritas no Apêndice deste guia intitulado Experiências Exitosas.

PARA SABER MAIS, CLIQUE NOS *LINKS* ABAIXO E/OU CONFIRA AS REFERÊNCIAS NO FINAL DO GUIA:

Política Nacional de Práticas Integrativas e Complementares no SUS (PNPIC)

Promoção da saúde e prevenção de doenças: SUS oferece 18 Práticas Integrativas

Outra estratégia que pode ser utilizada para redução do estresse de jovens estudantes por enfermeiros é a atenção plena ou *mindfulness*[2], que é um estado de consciência plena promovido a partir da escolha consistente e deliberada do indivíduo para tomar conhecimento do que está ocorrendo nos mundos interno e externo, exercitando a capacidade de estar completamente envolvido no momento presente, sem desviar sua atenção com eventos do passado ou do futuro (GHERARDI-DONATO et al, 2018).

[2] Para mais informações sobre o Mindfulness, acesse o artigo disponível em: https://periodicos.ufsm.br/reufsm/article/view/33058/pdf

Propostas de ações:

- Apoio emocional oferecendo tranquilidade, aceitação e encorajamento durante períodos de estresse por meio do acolhimento e escuta ativa;

- Apoio espiritual para que o jovem sinta equilíbrio e conexão com uma força maior discutindo sobre sistemas de crenças e visões de mundo e encorajando o uso de recursos espirituais, como oração e/ou visita a um conselheiro espiritual, conforme desejado;

- Assistência no controle da raiva facilitando a expressão desta de uma maneira adaptativa e não violenta utilizando uma abordagem calma e tranquilizadora, restringindo o acesso a armas em potencial e orientando sobre maneiras adequadas de expressão da raiva, técnicas de relaxamento e assertividade;

- Identificação e acompanhamento de estudantes com necessidades/problemas de saúde mental por meio da Consulta de Enfermagem;

- Encaminhamento para o(a) psicólogo(a) e/ou outros profissionais da RAS quando se tratar de uma situação que exija orientação/atendimento especializado, como casos graves de ansiedade e depressão;

- Facilitação da Aprendizagem por meio da promoção da capacidade de processar e compreender informações, considerando as necessidades e interesses dos jovens estudantes, utilizando materiais educativos apropriados e linguagem clara;

- Elaboração e implementação de programas educacionais com base nos princípios da saúde mental positiva (bem-estar psicológico e emocional) em parceria com professores e outros profissionais do IFCE ;

- Realização de grupos de apoio e terapêuticos (terapia comunitária integrativa) pelas equipes de assistência estudantil em parceria com outros profissionais/ serviços da comunidade, promovendo a liberdade de expressão e troca de experiências ;

- Ensino de técnicas de relaxamento, como Meditação, Técnicas de Respiração (respiração controlada), *Mindfulness* (atenção plena), Massagem, Reiki, Relaxamento por meio de imagens, Musicoterapia, entre outras;

- Estímulo de atividades físicas leves a moderadas, como caminhada, alongamento, Yoga e Tai chi chuan;

• Ensino de técnicas de organização do tempo e planejamento de estudo;

• Utilização da técnica de Entrevista Motivacional com foco no controle do estresse;

• Utilização de obras literárias, imagens e outros instrumentos mediadores para realização de atividades educativas com jovens;

• Parcerias com grupos/coletivos de jovens, como de música, dança e de produção de materiais artísticos (desenho e pintura) e audiovisuais;

• Estímulo de hábitos para um sono reparador, como ambiente apropriado, estabelecimento de horários para dormir e acordar, evitar alimentos pesados, bebidas estimulantes, álcool, fumo e uso de telas antes da hora de dormir;

• Promoção da Resiliência por meio da assistência a indivíduos, famílias e comunidades para o desenvolvimento, uso e fortalecimento de fatores protetores a serem utilizados para lidar com estressores ambientais e sociais;

• Criação/participação em grupos intersetoriais de discussão de ações de Saúde Mental no contexto do IFCE;

• Realização de atividades de educação em saúde sobre saúde mental e habilidades para vida em colaboração com a equipe multiprofissional de Assistência Estudantil;

• Implementação de programas de prevenção de transtornos mentais para jovens vulneráveis a condições de saúde mental como grupos minoritários ou discriminados (negros, mulheres, pessoas LGBTQIA+, entre outros);

• Oferta de métodos de autoajuda, incluindo intervenções eletrônicas de saúde mental.

• Identificação de estudantes com dificuldades de desenvolvimento e deficiências;

• Aconselhamento sobre o uso de tabaco, álcool e outras substâncias;

• Encaminhamento e apoio para gerenciamento de autoagressão e / ou risco de suicídio e outras condições psicossomáticas.

Observações:

- Embora as técnicas de gerenciamento de estresse apresentadas neste guia estejam dentro do escopo da prática do enfermeiro escolar, recomenda-se obter educação permanente para uso das referidas práticas;

- Uma série de abordagens diferentes para auxiliar indivíduos e famílias no gerenciamento do estresse foi apresentada a fim de familiarizar os enfermeiros com a gama de estratégias disponíveis. O estudante e o enfermeiro devem tomar a decisão sobre quais estratégias usar de forma colaborativa. Essa decisão deve ser baseada nas características do jovem, nas fontes de estresse experimentadas por ele e nos padrões gerais de resposta a eventos estressantes;

- Os enfermeiros são encorajados a consultar as referências no final deste guia para obter mais informações sobre o uso de estratégias de gerenciamento de estresse como intervenções de enfermagem.

PARA SABER MAI,S CLIQUE NO *LINK* ABAIXO E/OU CONFIRA AS REFERÊNCIAS NO FINAL DO GUIA:

[TRANSTORNOS DE ANSIEDADE: GUIA DE EDUCAÇÃO EM SAÚDE PARA ENFERMEIROS]

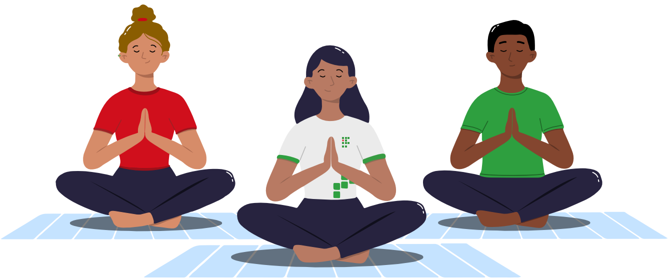

7.4 Estratégias de suporte social para promoção da saúde

O suporte social é considerado um processo recíproco e um recurso interativo que proporciona conforto, assistência, incentivo e informação (MURDAUGH; PARSONS; PENDER, 2019). Também é entendido como o conjunto de informações/percepções que levam um indivíduo a acreditar que é estimado, amado, cuidado e pertencente a uma rede com obrigações mútuas. O suporte social desempenha um papel importante na saúde e bem-estar. As relações sociais dos indivíduos são criticamente importantes na promoção da saúde.

Nesse contexto, as redes sociais são uma articulação intencional de pessoas /grupos como uma estratégia organizativa, que ajuda os agentes sociais a potencializar suas iniciativas para promover o desenvolvimento pessoal, acadêmico e profissional. Apesar de não se saber como realmente acontece, um ambiente social favorável pode promover a saúde e a qualidade de vida, além de reduzir os efeitos de doenças, principalmente aquelas ligadas aos sistemas neuroendócrino e imunológico (NAHAS, 2017).

Destacam-se as funções dos distintos grupos sociais na promoção da saúde, a família é considerada um grupo de suporte primário, outras fontes de suporte social são os pares, as organizações comunitárias e os grupos de autoajuda. O suporte social somente é efetivo quando existe um nível profundo de envolvimento e preocupação entre as pessoas. As qualidades críticas dentro de uma rede social são a troca de comunicações íntimas e a presença de solidariedade e confiança (SMELTZER et al., 2012).

O suporte social é uma possibilidade de atravessar as adversidades, conflitos e inseguranças inerentes ao processo de desenvolvimento dos jovens, promovendo a saúde e o bem-estar. Ele envolve aspectos cognitivos, sociais e afetivo-emocionais e busca alcançar a harmonia/motivação/sentido da vida, resultando em crescimento pessoal, empoderamento e autonomia. Inseridos em uma rede de relações os jovens contam com a ajuda das pessoas e instituições, tendem a estar mais protegidos nas situações de crises e vulnerabilidades, facilitando estratégias de enfrentamento e adaptação às mudanças.

A avaliação dos sistemas de suporte social precisa ser um componente básico da avaliação inicial de enfermagem usando estratégias como as descritas neste guia. Nessas avaliações, o enfermeiro deve saber como obter informações culturalmente sensíveis de diversas populações. Se as redes sociais precisam ser aprimoradas, o enfermeiro precisa desenvolver programas para ensinar aos estudantes as habilidades necessárias para desenvolver relacionamentos de apoio, prevenindo, assim, perdas de suporte social e solidão.

O enfermeiro deve considerar tanto o jovem quanto o meio social a fim de facilitar a promoção integral da saúde. O planejamento e a avaliação das intervenções de enfermagem para aumentar o suporte social são essenciais. Essas intervenções quando cientificamente sólidas podem melhorar a qualidade das relações humanas ao longo da vida.

Uma estratégia que pode ser utilizada por enfermeiros no contexto escolar é a educação entre pares com foco no protagonismo juvenil, valorizando a troca entre pessoas com experiências semelhantes. Nessa metodologia, os jovens atuam como facilitadores e multiplicadores de ações com seus pares, podendo realizar atividades como oficinas, atividades artísticas, rodas de conversas, dentre outras. Assim, viabilizando, desse modo, a elaboração

de estratégias críticas e artisticamente potentes para ações de caráter preventivo direcionadas a jovens em situação de vulnerabilidade social, fomentando a promoção da saúde no ambiente escolar (PADRÃO et al., 2021).

Finalmente, os enfermeiros precisam incorporar fontes de suporte social, como famílias, grupos de autoajuda, grupos da internet e de aplicativos de mensagens e organizações comunitárias em suas intervenções para aumentar o potencial de sucesso na promoção da saúde e mudança de estilo de vida dos jovens estudantes.

Propostas de ações:

- Obtenção de informações sobre as fontes de suporte social dos jovens estudantes;
- Realização de parcerias com instituições públicas e/ou privadas, organizações sociais, igrejas e associações comunitárias para apoiar o desenvolvimento das ações de saúde e assistência estudantil;
- Encaminhamento para o(a) assistente social e/ou outros profissionais/serviços da RAS quando se tratar de uma situação que exija orientação/atendimento especializado, como grande vulnerabilidade socioeconômica ou casos de violência;
- Divulgação/apresentação aos estudantes da rede de saúde disponível para a comunidade, na perspectiva da promoção, prevenção e tratamento dos principais agravos à saúde presentes na população;
- Incentivo ao diálogo entre profissionais de saúde/educação e estudantes;
- Promoção da convivência familiar e comunitária, auxiliando os jovens a ver suas famílias como um recurso para aconselhamento e apoio;
- Promoção do envolvimento familiar nos comportamentos de saúde dos jovens;
- Estímulo ao Protagonismo Juvenil por meio do envolvimento dos jovens em atividades escolares voluntárias, como participação nas entidades estudantis e ações extensionistas;
- Utilização do letramento em saúde para fornecer conhecimentos e habilidades aos jovens estudantes, estimulando comportamentos de saúde;
- Parcerias com professores e outros setores e profissionais do campus ligados ao ensino para motivar os jovens a perseguir conquistas e objetivos acadêmicos;
- Participação em reuniões de pais e mestres para aumentar a comunicação com a família e comunidade;
- Orientação aos pais sobre o processo de “adolescer” e a importância de sua participação na educação dos filhos;
- Realização de visitas domiciliares para estudantes com maior vulnerabilidade social, como gestantes, puérperas e pessoas com deficiência possibilitando uma maior aproximação e interação com a realidade socioeconômica e cultural dos alunos;
- Participação no programa de acolhida dos alunos ingressantes (seminário de integração dos

estudantes ao IFCE), apresentando o setor/ serviço de enfermagem aos estudantes;

- Criação/participação em grupos de jovens para educação entre pares sobre temas ligados à saúde;
- Encaminhamento e apoio para o manejo da gravidez, incluindo a opção para adolescentes grávidas ou pais continuarem ou retornarem à escola;
- Implantação de sala de apoio à amamentação nos campi para favorecer o aleitamento materno.

Observações:

Considerar as adolescências e as juventudes como construções históricas e sociais, identificando as situações de exclusão a que estão expostos os adolescentes e os jovens por meio de características como gênero, raça/etnia, orientação sexual e classe social;

Incorporar às práticas educativas e de saúde os elementos culturais presentes no território como uma forma de promover saúde por meio da valorização da cultura local.

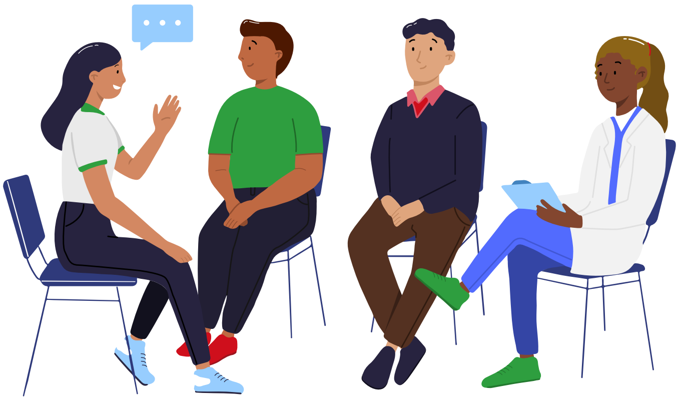

PARA SABER MAIS, CLIQUE NO *LINK* ABAIXO E/OU CONFIRA AS REFERÊNCIAS NO FINAL DO GUIA:

[Redes de apoio ao adolescente no contexto do cuidado à saúde: interface entre saúde, família e educação]

8. COMO E QUANDO UTILIZAR O GUIA?

Este Guia como ferramenta orientativa oferece informações estruturadas sobre estratégias de promoção da saúde que poderão ser desenvolvidas com jovens estudantes. Assim, o enfermeiro pode buscar informações para tomada de decisão conforme a demanda da sua prática assistencial em uma ou mais dessas estratégias, considerando sua expertise, as condições da instituição de ensino e as preferências do estudante para que este assuma um papel ativo na busca da saúde e do autocuidado.

Os enfermeiros escolares podem implementar as estratégias e ações propostas pelo guia com estudantes, grupos e famílias, nas escolas e comunidade em geral, durante a consulta de enfermagem e/ou em outros momentos em que ocorra o encontro com jovens, como em sala de aula e outras atividades acadêmicas, buscando não perder nenhuma oportunidade. Eles devem atuar com vistas ao fortalecimento do autocuidado e favorecendo o bem-estar do estudante por meio da educação e desenvolvimento pessoal.

Ressalta-se, ainda, que no guia somente foram abordados os quatro domínios de promoção da saúde propostos pelo MPS, que são atividade física, alimentação saudável, gerenciamento do estresse e suporte social. No entanto, há outras temáticas relacionadas à promoção da saúde que podem ser desenvolvidas pelos enfermeiros no contexto escolar, como a saúde sexual e reprodutiva, uso de substâncias, Doenças Crônicas Não-Transmissíveis (DCNT), dentre outras, por serem necessárias a essa população.

Acredita-se que, por meio dessa tecnologia, é possível fornecer informações relevantes que possam contribuir também com a mudança de comportamentos de saúde dos jovens estudantes, fortalecendo a sua autonomia e responsabilidade, ajudando na promoção da saúde. Assim, a utilização desse material pode melhorar a prática educativa do enfermeiro e da equipe de assistência estudantil.

9. FATORES FACILITADORES E BARREIRAS PARA UTILIZAÇÃO DO GUIA

Apresentamos a seguir alguns fatores identificados pelos enfermeiros do IFCE, participantes dos grupos focais para elaboração do guia, os quais foram organizados como facilidades e barreiras na implementação das estratégias de promoção da saúde com jovens estudantes propostas pelo mesmo.

Destaca-se que o processo de mudanças e/ou inovações na assistência de enfermagem e saúde caracteriza-se como um trabalho coletivo. Desse modo, faz-se necessário buscar acordos e apoio para mudança/inovação com os atores sociais atuantes no contexto (profissionais, estudantes e gestores) para alcançar seu comprometimento. Esse processo requer tempo, paciência e persistência.

Nesse sentido, o planejamento estratégico e a educação permanente em saúde podem ajudar os enfermeiros e demais profissionais do IFCE a alcançar as competências únicas exigidas para os desafios de um trabalho integrado entre educação e saúde. Uma compreensão abrangente das necessidades de aprendizagem dos enfermeiros é necessária para orientar o cuidado clínico e o desenvolvimento da equipe.

Quadro 1 – Fatores facilitadores e barreiras para utilização do Guia

FACILITADORES	BARREIRAS
• Abrangência do MPS<br>• Planejamento integrado com o setor de ensino<br>• Garantia de espaço/tempo para atividades de educação/promoção da saúde no Projeto Pedagógico do Curso (PPC) e/ou no calendário acadêmico do *campi*<br>• Campanhas com materiais visuais nas redes sociais<br>• Trabalho com grupos e líderes de pares<br>• Certificado de participação nas ações, premiações e entrega de brindes<br>• Participação e engajamento dos docentes nas ações de assistência estudantil/promoção da saúde<br>• Interesse dos jovens por testes, exames laboratoriais e avaliações clínicas<br>• Sistematização das ações com embasamento teórico<br>• Colaboração interprofissional reconhecendo os limites de atuação de cada categoria<br>• Pesquisa com os estudantes para sugestões de temas das ações<br>• Esclarecer o papel do enfermeiro escolar e sua importância<br>• Interesse/desejo dos profissionais de realizar atividades educativas com o público jovem<br>• Uso das Práticas Integrativas e Complementares em Saúde (PICS)<br>• Boa estrutura física do IFCE para a prática de atividade física<br>• Organização de espaços de planejamento, ação e avaliação compartilhadas entre profissionais da educação e saúde	• Aplicabilidade prática dos componentes do MPS<br>• Fragmentação das ações<br>• Carga horária elevada dos cursos, principalmente do técnico integrado<br>• Diferenças geracionais, culturais e sociais entre profissionais e estudantes<br>• Pequena participação e desinteresse dos estudantes nas ações de educação em saúde<br>• Desmotivação e falta de engajamento institucional dos profissionais<br>• Despreparo/dificuldade dos profissionais para atuar no ambiente escolar e/ou com jovens<br>• Falta de apoio da gestão<br>• Escassez de recursos materiais, audiovisuais e financeiros<br>• Articulação com a rede de saúde dos municípios<br>• Foco no atendimento clínico e por demanda espontânea de queixas físicas<br>• Centralização das ações da assistência estudantil na concessão de auxílios financeiros<br>• Grande oferta de alimentos processados nos lanches e refeições e resistência dos estudantes para consumir alimentos naturais<br>• Poucas oportunidades durante a formação e educação permanente para enfermeiros com foco na nutrição e alimentação saudável<br>• Pequena participação de enfermeiros em ações de promoção da alimentação saudável<br>• Resistência de alguns alunos em participar das aulas de educação física, principalmente os ingressantes<br>• Alto custo financeiro e falta de apoio da gestão para participação dos estudantes nos Jogos do IFCE<br>• Ausência de perspectiva de futuro ou projeto de vida por parte de alguns jovens<br>• Descontextualização da instituição de ensino com a realidade socioeconômica dos estudantes

10. DIFUSÃO E DISSEMINAÇÃO DO GUIA DE ENFERMAGEM ESCOLAR

A seguir, apresentamos algumas ações que podem ser realizadas pela Equipe de Enfermagem do IFCE para promover a difusão e disseminação do Guia e, consequentemente, sua utilização na prática profissional.

- Divulgar o Guia nas reuniões com os gestores e profissionais da Assistência Estudantil do IFCE;

- Incluir a utilização do Guia como uma ação do Plano de Desenvolvimento Institucional (PDI), Plano Anual de Ações (PAA) e/ou Cronograma Sistêmico de Atividades da Diretoria de Assuntos Estudantis (DAE) e da Equipe de Enfermagem do IFCE;

- Incluir as informações do guia na programação dos encontros pedagógicos dos *campi*, para que as comunidades acadêmicas tomem conhecimento, sensibilizem-se e apoiem as ações de promoção da saúde com estudantes;

- Incluir a divulgação do Guia nas publicações oficiais impressas e digitais/eletrônicas do IFCE (boletins, informes, notícias nas redes sociais, entre outros);

- Incluir na programação do Fórum de Assuntos Estudantis o tema promoção da saúde com base nas informações do Guia;

- Fomentar a inclusão das orientações do Guia nas diversas ações da Equipe de Enfermagem e assistência estudantil;

- Promover ações educativas (palestras, rodas de conversa, debates, cursos e outras) com os estudantes e os profissionais de saúde com base nas orientações do Guia;

- Estimular a inclusão das orientações do Guia nos programas de assistência estudantil, promoção da saúde, promoção da atividade física, promoção da alimentação saudável, controle do estresse e nas ações de suporte psicossocial já existentes no IFCE;

- Utilizar os conteúdos do Guia em ações, eventos e campanhas regulares já realizadas pelas equipes de enfermagem e assistência estudantil –Dia Mundial da Saúde (7 de abril), prevenção ao suicídio (Setembro Amarelo), saúde da mulher (Outubro Rosa), saúde do homem (Novembro Azul) e campanhas de vacinação, entre outros, além de estimular que os profissionais os utilizem em consultas de rotina, grupos já existentes, visitas domiciliares e em programas ofertados pela equipe e outros espaços já frequentados pelos estudantes.

- Promover a participação dos estudantes na avaliação dos conteúdos do guia;

- Construir outros materiais educativos a partir do Guia, como cartilhas, fôlderes e jogos.

REFERÊNCIAS

ANTUNES, P. de C.; LAGRANHA, D.M.; SOUSA, M.F. de; SILVA, A.M.; FRAGA, A.B. Revisão sistemática sobre práticas corporais na perspectiva das práticas integrativas e complementares em saúde. **Motrivivência**, Florianópolis, v. 30, n. 55, p. 227-247, set. 2018. Disponível em: https://doi.org/10.5007/2175-8042.2018v30n55p227 Acesso em: 10 set. 2021.

BEST, N.C.; OPPEWAL, S.; TRAVERS, D. Exploring School Nurse Interventions and Health and Education Outcomes: An Integrative Review. **The Journal of School Nursing**, Silver Spring, v.34, n.1, p. 14–27, 2018. Disponível em: https://doi.org/10.1177/1059840517745359. Acesso em: 05 ago. 2021.

BITTENCOURT, M.N.; MARQUES, M.I.D.; BARROSO, T.M.M.D.A. Contributos das teorias de enfermagem na prática da promoção de saúde mental. **Revista de Enfermagem Referência**, Coimbra, série IV, n.º 18, p. 125 - 132, jul. 2018. Disponível em: https://doi.org/10.12707/RIV18015. Acesso em: 12 jan. 2022.

BRASIL. **Lei nº 7.498/86, de 25 de junho de 1986**. Dispõe sobre a regulamentação do exercício da enfermagem, e dá outras providências. Diário Oficial da União. Brasília, DF, 26 jun. 1986. Disponível em: http://www.planalto.gov.br/ccivil_03/leis/L7498.htm. Acesso em: 12 ago 2021.

BRASIL. **Lei nº 11.892, de 29 de dezembro de 2008**. Institui a Rede Federal de Educação Profissional, Científica e Tecnológica e cria os Institutos Federais de Educação, Ciência e Tecnologia. Brasília: Diário Oficial da União, 2008.

BRASIL. Ministério da Saúde. **Caderno do gestor do PSE**. Brasília: Ministério da Saúde, 2015a.

BRASIL. Ministério da Saúde. Ministério da Educação. **Guia de sugestões de atividades:** Semana Saúde na Escola 2015 (versão preliminar). Brasília: Ministério da Saúde, 2015b. Disponível em: http://189.28.128.100/dab/docs/portaldab/documentos/guia_atividades_pse_versao_preliminar.pdf. Acesso em: 26 jul. 2021.

BRASIL. Ministério da Saúde. Secretaria de Atenção à Saúde. Departamento de Atenção Básica. **Guia alimentar para a população brasileira**. 2. ed., 1. reimpr. Brasília: Ministério da Saúde, 2014. Disponível em: https://bvsms.saude.gov.br/bvs/publicacoes/guia_alimentar_populacao_brasileira_2ed.pdf. Acesso em: 27 jul. 2021.

BRASIL. Ministério da Saúde. Secretaria de Atenção à Saúde. Departamento de Atenção Básica. **Política nacional de práticas integrativas e complementares no SUS:** atitude de ampliação de acesso. 2. ed. Brasília: Ministério da Saúde, 2015c.

BRASIL. Ministério da Saúde. Secretaria de Atenção à Saúde. Departamento de Atenção Básica. **Programa Saúde na Escola:** Caderno Temático Práticas Corporais, Atividade Física e Lazer (versão preliminar). Brasília: Ministério da Saúde, 2015d. Disponível em: http://189.28.128.100/dab/docs/

portaldab/documentos/caderno_praticas_corporais_atividade-fisica_lazer.pdf. Acesso em: 20 jul. 2021.

BRASIL. Ministério da Saúde. Secretaria de Atenção à Saúde. Departamento de Atenção Básica. **Programa Saúde na Escola:** Caderno Temático Segurança Alimentar e Nutricional e Promoção da Alimentação Adequada e Saudável (versão preliminar). Brasília: Ministério da Saúde, 2015e. Disponível em: http://189.28.128.100/dab/docs/portaldab/documentos/caderno_san_paas.pdf. Acesso em: 21 jul. 2021.

BRASIL. Ministério da Saúde. Secretaria de Atenção Primária à Saúde. Departamento de Promoção da Saúde. **Guia de Atividade Física para a População Brasileira** [recurso eletrônico]. Brasília: Ministério da Saúde, 2021a. Disponível em: https://bvsms.saude.gov.br/bvs/publicacoes/guia_atividade_fisica_populacao_brasileira.pdf Acesso em 27 jul. 2021.

BRASIL. Ministério da Saúde. Secretaria de Atenção Primária à Saúde. Departamento de Promoção da Saúde. **Guia de Atividade Física Para a População Brasileira:** recomendações para gestores e profissionais de saúde [recurso eletrônico]. Brasília: Ministério da Saúde, 2021b. Disponível em: http://189.28.128.100/dab/docs/portaldab/publicacoes/guia_atv_gestores.pdf. Acesso em: 27 jul. 2021.

BRASIL. Ministério da Saúde. Secretaria-Executiva. Secretaria de Atenção à Saúde. **Glossário temático:** práticas integrativas e complementares em saúde. Brasília: Ministério da Saúde, 2018. Disponível em: http://189.28.128.100/dab/docs/portaldab/publicacoes/glossario_pics.pdf Acesso em: 27 jul. 2021

BULECHEK G.M.; BUTCHER H.K.; DOCHTERMAN J.M.; WAGNER, C.M. [tradução de Denise Costa Rodrigues]. **Classificação das Intervenções em Enfermagem (NIC).** 6 ed. Rio de Janeiro: Elsevier; 2016.

COCKERHAM, W. C. Health Behavior. In: COCKERHAM, W. C.; DINGWALL, R.; QUAH, S. **The Wiley Blackwell Encyclopedia of Health, Illness, Behavior, and Society [Internet].** 2014. Disponível em: https://onlinelibrary.wiley.com/doi/abs/10.1002/9781118410868.wbehibs296. Acesso em: 25 nov .2019. doi:10.1002/9781118410868.wbehibs296

CONSELHO FEDERAL DE ENFERMAGEM. **Resolução COFEN Nº 358/2009**. Brasília: COFEN, 2017. Disponível em: http://www.cofen.gov.br/resoluo-cofen-3582009_4384.html. Acesso em: 22 jul. 2021.

CONSELHO REGIONAL DE ENFERMAGEM DE SÃO PAULO. **Processo de enfermagem:** guia para a prática. 2.ed. São Paulo: COREN-SP, 2021. Disponível em: https://portal.coren-sp.gov.br/sites/default/files/SAE-web.pdf. Acesso em: 18 jan. 2022.

COSTA, R.F. da et al. Redes de apoio ao adolescente no contexto do cuidado à saúde: interface entre saúde, família e educação. **Revista da Escola de Enfermagem da USP**, São Paulo, v. 49, n. 5, p. 741-747, out. 2015. Disponível em: https://doi.org/10.1590/S0080-623420150000500005. Acesso em: 25 mai 2018.

DICK, B. FERGUSON, B.J. Health for the World's Adolescents: A Second Chance in the Second Decade. **Journal of Adolescent Health**, Philadelphia, v. 56, p.3-6, 2015. Disponível em: http://dx.doi.org/10.1016/j.jadohealth.2014.10.260 Acesso em: 13 mar. 2019

FIGLIE, N.B.; GUIMARAES, L.P. A Entrevista Motivacional: conversas sobre mudança. **Boletim - Academia Paulista de Psicologia**, São Paulo, v. 34, n. 87, p. 472-489, dez. 2014. Disponível em: http://pepsic.bvsalud.org/pdf/bapp/v34n87/a11.pdf Acesso em: 27 jul. 2021.

GHERARDI-DONATO, E.C.S.; FERNANDES, M.N.F.; SCORSOLINI-COMIN, F.; ZANETTI, A.C.G. Mindfulness: reflexão sobre limites e potencialidades para a assistência de enfermagem. **Revista de Enfermagem da UFSM**, Santa Maria, v.9 n.21, jul. 2019. Disponível em: https://periodicos.ufsm.br/reufsm/article/view/33058/pdf Acesso em 28 jul. 2021.

GUEDES, D.P. ZUPPA, M.A. Propriedades Psicométricas da Versão Brasileira da Adolescent Health Promotion Scale (AHPS). **Ciência e Saúde Coletiva** , Rio de Janeiro, v.25, n.6, jun. 2020. Disponível em:https://doi.org/10.1590/1413-81232020256.23252018. Acesso em: 06 jan. 2022.

INSTITUTO FEDERAL DO CEARÁ. **A presença do IFCE no Ceará**. Disponível em: https://ifce.edu.br/acesso-rapido/campi/campi. Acesso em: 25 set. 2019.

INSTITUTO FEDERAL DO CEARA. **IFCE em números**. IFCE, 2021a. Disponível em: http://ifceemnumeros.ifce.edu.br/. Acesso em: 27 jul. 2021.

INSTITUTO FEDERAL DO CEARÁ. **Projeto político-pedagógico institucional/ Instituto Federal do Ceará**. Fortaleza: IFCE, 2018. 152 p.

INSTITUTO FEDERAL DE EDUCAÇÃO, CIÊNCIA E TECNOLOGIA DO CEARÁ. Campus Avançado de Jaguaruana. PEREIRA, F.W (organizadora). **Energia positiva:** inserindo práticas integrativas e complementares no seu dia a dia em tempos de coronavírus e até quando você se permitir! Jaguaruana: IFCE, 2020. Disponível em: https://ifce.edu.br/noticias/cartilha-orienta-sobre-saude-fisica-e-emocional/copy_of_CARTILHAENERGIAPOSITIVA1.pdf Acesso em 23 Jun. 2021.

INSTITUTO FEDERAL DE EDUCAÇÃO, CIÊNCIA E TECNOLOGIA DO CEARÁ. Campus de Morada Nova. Departamento de Ensino. Setor de Assistência Estudantil. **Projeto Girassol:** acolher e cuidar. Morada Nova: IFCE, 2021b.

INSTITUTO FEDERAL DO CEARA. Diretoria de Assuntos Estudantis – DAE. **Referenciais de atuação dos Profissionais da Assistência Estudantil.** v.1. Fortaleza: IFCE, 2016. Disponível em: https://ifce.edu.br/espaco-estudante/assistencia-estudantil/publicacoes/referenciais-de-atuacao-dos-profissionais-da-ae.pdf/view. Acesso em: 18 jan. 2022.

INSTITUTO FEDERAL DO CEARÁ. Pró-reitoria de Ensino. **Plano Estratégico para Permanência e Êxito dos Estudantes do IFCE**/ Armênia Chaves Fernandes Vieira, Erica de Lima Gallindo, Hobson Almeida Cruz. Fortaleza: IFCE, 2017.

INTERNATIONAL COUNCIL OF NURSES. **Classificação Internacional para a Prática de Enfermagem (CIPE®)** – Versão 2017 – Português do Brasil. Disponível em: https://www.icn.ch/sites/default/files/inline-files/icnp-brazil-portuguese-translation-2017.pdf Acesso em: 23 jul. 2021.

LIMA, L.F. de A. **Transtornos de ansiedade:** guia de educação em saúde para enfermeiros. Fortaleza, CE: Ed. da Autora, 2021. Disponível em: http://sanusb.org/arquivos/ebook_guia_ansiedade.pdf. Acesso em: 18 jan. 2022.

LINEBERRY, M.J.; ICKES, M.J. The Role and Impact of Nurses in American Elementary Schools: A Systematic Review of the Research. **The Journal of School Nursing,** Silver Spring, v. 31, n.1, p. 22-33, 2015. Disponível em: https://doi.org/10.1177/1059840514540940. Acesso em: 27 dez. 2018.

MONTEIRO, P. de V. **Programa de intervenções educativas para prevenção de IST/HIV entre adolescentes e jovens.** Programa de Pós-graduação Cuidados Clínicos, Enfermagem e Saúde (tese). Universidade Estadual do Ceará: Fortaleza, 2017 (154p.). Disponível em: https://siduece.uece.br/siduece/trabalhoAcademicoPublico.jsf?id=84477 Acesso em: 05 ago. 2021.

MUNIZ, E.A.; QUEIROZ, M.V.O.; DUTRA, F.C.S.; ARAÚJO, A.F.; SILVA, L.M.S.; TORRES, R.A.M. Políticas de saúde e educação para a juventude no Brasil: intersetorialidade e atuação do enfermeiro. **Sanare**, Sobral, v.20, n.1, p.73-80, 2021. Disponível em: https://doi.org/10.36925/sanare.v20i1.1552. Acesso em: 18 jan. 2022.

MURDAUGH, C.L.; PARSONS, M.A.; PENDER, N.J. **Health Promotion in Nursing Practice**. 8 ed. New York: Pearson, 2019.

NACIONAL ASSOCIATION OF SCHOOL NURSES. **Position Statements, Position Briefs, Joint Statements**. Silver Spring: NASN, 2018.

NAHAS, M.V. A**tividade física, saúde e qualidade de vida:** conceitos e sugestões para um estilo de vida ativo. 7. ed. Florianópolis: Ed. do Autor, 2017.

ORGANIZAÇÃO PANAMERICANA DE SAÚDE (OPAS). **Saúde mental dos adolescentes** [Internet]. OPAS, 2021. Disponível em: https://www.paho.org/pt/topicos/saude-mental-dos-adolescentes. Acesso em: 26 jul. 2021.

ORGANIZAÇÃO PANAMERICANA DE SAÚDE (OPAS). **Mitigação das consequências diretas e indiretas da COVID-19 sobre a saúde e o bem-estar dos jovens nas Américas**. Washington: OPAS, 2021. Disponível em: https://iris.paho.org/bitstream/handle/10665.2/55304/OPASFPLHLCOVID-19210037_por.pdf?sequence=1&isAllowed=y. Acesso em 10 jan. 2022.

PADRÃO, M.R.A. de V. et al. Educação entre pares: protagonismo juvenil na abordagem preventiva de álcool e outras drogas. **Ciência & Saúde Coletiva**, Rio de Janeiro, v. 26, n. 07, p. 2759-2768, 2021. Disponível em: https://doi.org/10.1590/1413-81232021267.07322021. Acesso em: 14 set 2021.

PAN AMERICAN HEALTH ORGANIZATION. **The Burden of Mental Disorders in the Region of the Americas**, 2018. Washington: PAHO, 2018.

PATTON, G.C. et al. Our future: a Lancet commission on adolescent health and wellbeing. **Lancet**, London, v. 387, p. 2423–2478, 2016. Disponível em: http://dx.doi.org/10.1016/S0140-6736(16)00579-1 Acesso em: 13 mar. 2019.

PEARSON, A.; WEEKS, S.; STERN C. **Translation science and the JBI model of evidence based healthcare.** Philadelphia: Lippincott Williams & Wilkins, 2011.

PEDUZZI, M.; AGRELI, H.F. Trabalho em equipe e prática colaborativa na Atenção Primária à Saúde. **Interface - Comunicação, Saúde, Educação**, Botucatu , v. 22, n. supl. 2, p. 1525-1534, 2018. Disponível em: https://doi.org/10.1590/1807-57622017.0827. Acesso em: 12 jan. 2022.

SANTOS, A.S. et al. Tecnologia educacional baseada em Nola Pender: promoção da saúde do adolescente. **Revista de Enfermagem UFPE on line**, Recife, v.12, n.2, p.582-588, fev. 2018. Disponível em: https://doi.org/10.5205/1981-8963-v12i2a22609p582-588-2018. Acesso em: 05 ago. 2021.

SMELTZER, S.C.; BARE, B.G.; HINKLE, J.L.; CHEEVER, K.H. Brunner & Suddarth – **Tratado de Enfermagem Médico-Cirúrgica.** 12 ed. v.1. Rio de Janeiro: Guanabara Koogan, 2012.

SOBRINHO, A. et al. Condição Juvenil na América Latina: demandas e enfrentamentos às iniquidades em saúde. **Ciência & Saúde Coletiva**, Rio de Janeiro, v. 26, n. 7, p. 2562, 2021. Disponível em: https://doi.org/10.1590/1413-81232021267.08262021. Acesso em: 30 ago. 2021.

WORLD HEALTH ORGANIZATION. UNITED NATIONS EDUCATIONAL, SCIENTIFIC AND CULTURAL ORGANIZATION. **WHO guideline on school health services**. Geneva: World Health Organization, 2021.

EXPERIÊNCIAS EXITOSAS EM PRÁTICAS DE PROMOÇÃO DA SAÚDE PELA EQUIPE DE ENFERMAGEM DO IFCE

Apresentamos a seguir algumas experiências exitosas de cuidados de enfermagem e atividades educativas desenvolvidas pela Equipe de Enfermagem do IFCE com foco na promoção da saúde dos jovens estudantes.

1. Projeto de extensão "Protagonismo juvenil em cena: enfoques da promoção da saúde"

Sob a coordenação da enfermeira Ítala Dias, construíram-se vídeos educativos em formato de documentário com participação ativa de adolescentes por meio de histórias de vida e superação e utilizando os recursos de autoeficácia. Os vídeos abordavam temáticas de interesse dos estudantes identificadas por meio de questionários como *bullying*, acidentes de trânsito, arboviroses, Infecções Sexualmente Transmissíveis (IST), alimentação saudável, atividade física, gravidez na adolescência, saúde mental e diversidade de gênero, raça e orientação sexual. Os vídeos foram exibidos para adolescentes em escolas públicas de Tauá – CE e, posteriormente, disponibilizados nas redes sociais do IFCE - *Campus* Tauá. A ação propiciou aos adolescentes obter acesso a informações relevantes em torno dos principais agravos à saúde, refletir em torno dos comportamentos e riscos envolvendo as temáticas abordadas, esclarecer dúvidas, expressar opiniões e sentimentos.

PARA SABER MAIS SOBRE O PROJETO, ACESSE O ARTIGO POR MEIO DO *LINK*:

Utilização de vídeos explicativos para promoção da saúde do adolescente - uma ação de extensão do IFCE Campus Tauá

2. Circuito da saúde

O Circuito da Saúde é uma ação educativa que ocorre anualmente no mês de abril, no IFCE - *Campus* Crateús, organizado pela equipe de Assistência Estudantil, com o objetivo de oferecer ações e serviços à comunidade interna e externa de avaliação do estado geral de saúde e fornecimento de informações sobre a importância do autocuidado. Além de promover atividades esportivas e de lazer que incentivem a adoção de um estilo de vida ativo e fortalecer as parcerias com a rede assistencial do município por meio de instituições públicas e privadas.

Desde sua primeira edição em 2015, inclui na programação a oferta de serviços básicos em saúde como vacinação, aferição de medidas antropométricas e sinais vitais, cadastramento do cartão do SUS, testes rápidos para ISTs, doação de sangue, além de oficinas, passeio ciclístico, corrida, campanhas e palestras com temas relevantes para saúde. A cada ano, tem-se priorizado algum tema de acordo com as questões de saúde coletiva vivenciada pela comunidade acadêmica, como drogas, alimentação saudável e qualidade de vida. A ação vem crescendo e contando com uma adesão cada vez maior da comunidade.

O evento tem seu caráter extensionista ressaltado ao proporcionar a visitação e utilização dos serviços do *campus* pela comunidade externa. A ação tem possibilitado ao IFCE aproximar-se das instituições atuantes na região, o que facilita a construção de outras parcerias e fluxos de encaminhamentos da equipe para as redes assistenciais e tornando-se atividade fixa do calendário acadêmico.

3. Terapia Reiki

O Reiki é considerado um método voltado ao restabelecimento do sistema energético corporal, graças ao estímulo dos processos de cura natural do organismo, que pode ser utilizado para induzir o relaxamento e tratar problemas de saúde. É uma Prática Integrativa e Complementar em Saúde (PICS) reconhecida pelo Sistema Único de Saúde (SUS) e que vem sendo utilizada pela Enfermagem. Considerado uma abordagem holística para a saúde e bem-estar, o Reiki também pode ser entendido como uma filosofia de vida que promove equilíbrio e felicidade.

As PICS estão sendo desenvolvidas por meio de um projeto de qualidade de vida e saúde coordenado pela enfermeira e mestre em Reiki do *campus* Avançado de Jaguaruana, Fabiani Pereira. O projeto iniciou com a realização da anamnese espiritual de estudantes e servidores durante a Consulta de Enfermagem. Ao fazer isso, a enfermeira constatou a necessidade de ofertar uma atividade educativa que atenda a essa necessidade humana. Assim, realizou um projeto com o intuito de abordar a saúde em sua totalidade, iniciando o método Reiki em seu contexto de trabalho, por meio de uma agenda programada duas vezes por semana em turnos diferenciados, totalizando 10 atendimentos por semana.

Após realizar uma atividade educativa com um grupo de estudantes e comunidade externa sobre 28 dias de prática da lei da atração, o Reiki também foi difundido para a comunidade externa, principalmente aos familiares dos estudantes.

O método Reiki como prática unitária e terapêutica tem evidenciado ótimos resultados entre estudantes, servidores, comunidade externa e para a enfermeira que aplica o método. Ele exige poucos recursos materiais para sua execução e raramente foi utilizado como prática única, pois as pessoas costumam retornar ao ambulatório de enfermagem e encaram o Reiki como uma terapia, já que elas recebem no mínimo entre duas e quatro sessões. Os resultados são benéficos e promissores, sendo uma terapia que merece ser valorizada e disseminada para outros *campi* e instituições.

4. Auriculoterapia

A Auriculoterapia é uma técnica milenar, baseada em conhecimentos tradicionais, que reconhece o pavilhão auricular como um microssistema e zona reflexa ligados a determinadas estruturas e órgãos do corpo. Na técnica, utilizam-se pontos de acordo com as queixas identificadas na anamnese e, ao serem estimulados, provocam reações neuroendócrinas que auxiliarão no reparo terapêutico.

As práticas são realizadas pela enfermeira do *Campus* de Morada Nova Kaline Freitas, que possui certificação comprovada em estudantes e servidores com problemas mentais, principalmente ansiedade. Assim, o ambulatório do *campus* é utilizado para a construção de protocolos, de saberes e de práticas. A

auriculoterapia aumentou a visibilidade e a demanda de atendimentos da enfermeira com possibilidade de respostas às necessidades dos estudantes.

5. Elaboração de materiais educativos

A Equipe de Enfermagem do IFCE vem elaborando diversos materiais educativos e audiovisuais, como *cards*, *quiz*, vídeos e cartilhas sobre temas relacionados à saúde para divulgação nas redes sociais da instituição. Essas atividades foram intensificadas durante a pandemia da covid-19 em função do isolamento social e do ensino/trabalho remoto. Alguns exemplos são apresentados a seguir:

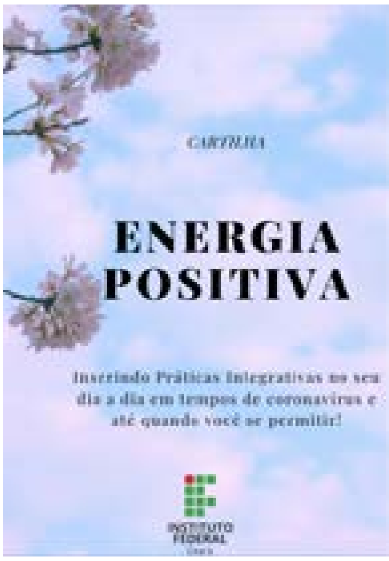

Fonte: IFCE - *Campus* Avançado de Jaguaruana, 2020.

- **Cartilha "Energia positiva: inserindo práticas integrativas e complementares no seu dia a dia em tempos de coronavírus e até quando você se permitir!"**, organizada pela enfermeira Fabiani Pereira do IFCE - *Campus* Avançado de Jaguaruana. A cartilha possui orientações simples, práticas e acessíveis que objetivam contribuir para o fortalecimento do autocuidado por meio da inserção de algumas práticas integrativas no cotidiano. Embora tenha emergido no contexto da pandemia de covid-19, pode ser utilizada para além dela, enfocando a importância da busca contínua do equilíbrio entre mente, corpo e espírito.

Acesse a cartilha aqui

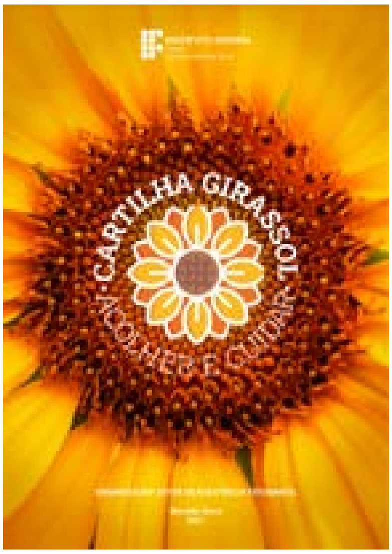

Fonte: IFCE - *Campus* Morada Nova, 2021.

- **Cartilha "Projeto Girassol: acolher e cuidar"**, organizada pelo Setor de Assistência Estudantil do IFCE – *Campus* Morada Nova. O Projeto Girassol utilizou as Práticas Integrativas e Complementares (PICS) - métodos de tratamentos milenares baseados em conhecimentos tradicionais - como forma de promoção, prevenção e recuperação da saúde, contribuindo para uma melhor qualidade de vida e bem-estar. O objetivo principal do projeto é colaborar e agregar novas formas de cuidado em saúde com ênfase no autocuidado. A cartilha aborda diversas formas de autocuidado com orientações simples, imagens ilustrativas dos próprios autores, que somaram aos seus conhecimentos amor, dedicação e compromisso.

Acesse a cartilha aqui

CONTATOS DOS SERVIÇOS DE ENFERMAGEM DO IFCE

Acaraú

Emanoel Avelar Muniz - Enfermeiro

enfermagem.acarau@ifce.edu.br

emanoel.muniz@ifce.edu.br

Aracati

Marcela Lima Silveira Praxedes - Enfermeira

cae.aracati@ifce.edu.br

Quezia Melo Martins - Técnica de Enfermagem

quezia.melo@ifce.edu.br

Baturité

Angélica Maria de Oliveira Almeida

angelica.almeida@ifce.edu.br

Boa Viagem

Keedi Jane Barbosa de Albuquerque Silva - Enfermeira

keedi.barbosa@ifce.edu.br

Camocim

Flávia Marques Xavier - Técnica de Enfermagem

sae.camocim@ifce.edu.br

Canindé

Edmara Teixeira Oliveira - Enfermeira

Antonia Edinaria da Silva - Técnica de enfermagem

cae.caninde@ifce.edu.br

Caucaia

Nayara Sousa de Mesquita - Enfermeira

nayara.sousa@ifce.edu.br

Sandro Mário Gurgel de Freitas

sandro.freitas@ifce.edu.br

Damiana Vieira Sampaio - Técnica de Enfermagem

damianasampaio@ifce.edu.br

Cedro

Jadna Mony Gregorio Freitas - Enfermeira

jadna.freitas@ifce.edu.br

Mirlene Alves Cavalcante - Técnica de Enfermagem

saude.cedro@ifce.edu.br

Crateús

Francicleide Geremias da Costa Souza - Técnica de Enfermagem

francicleide.geremias@ifce.edu.br

Crato

Kamille Ribeiro Sampaio - Enfermeira

kamille.r.sampaio@ifce.edu.br

Nyagra Ribeiro de Araújo - Enfermeira

nyagra.ribeiro@ifce.edu.br

Fortaleza

Cleane Chaves Maia - Enfermeira

cleane.maia@ifce.edu.br

Francileudo Santos de Abreu - Enfermeiro

francileudo.abreu@ifce.edu.br

Francisco Rogerlândio Martins de Melo - Enfermeiro

rogerlandio.martins@ifce.edu.br

Iguatu

Itala Keane Rodrigues Dias - Enfermeira

itala.rodrigues@ifce.edu.br

Maria Nezeneide Carneiro de Oliveira - Auxiliar de Enfermagem

neidecarneiro@ifce.edu.br

Itapipoca

Paulo Cesar Teles Correia Júnior - Enfermeiro

paulo.teles@ifce.edu.br

Narcelio Candido de Moura Júnior - Técnico de Enfermagem

narcelio.junior@ifce.edu.br

Jaguaruana

Fabiani Weiss Pereira - Enfermeira

fabiani.weiss@ifce.edu.br

Juazeiro do Norte

Francisco Lindomar Gomes Fernandes - Enfermeiro

francisco.fernandes@ifce.edu.br

Limoeiro do Norte

Carla Lidiany Bezerra Silva Oliveira - Enfermeira

senf@limoeiro.ifce.edu.br

Maracanaú

Agnes Caroline Souza Pinto - Enfermeira

cae.maracanau@ifce.edu.br

Lucélia Fernandes de Almeida Lima - Técnica de Enfermagem

lucelia.fernandes@ifce.edu.br

Morada Nova

Kaline Ribeiro de Freitas - Enfermeira

kaline.ribeiro@ifce.edu.br

Paracuru

Milena Leite Albano - Enfermeira

cae.paracuru@ifce.edu.br

Pecém

Elisângela Alves do Nascimento - Técnica de Enfermagem

elisangela.alves@ifce.edu.br

Quixadá

Denise Tomaz Aguiar - Enfermeira

denise.tomaz@ifce.edu.br

Sobral

Aldiania Carlos Balbino - Enfermeira

aldiania.balbino@ifce.edu.br

Tabuleiro do Norte

Persia Regilda Maia Rebouças - Enfermeira

persia.reboucas@ifce.edu.br

Tauá

Sharlene Pereira Alves - Enfermeira

saest.taua@ifce.edu.br

Ubajara

Diego de Sousa Pontes - Enfermeiro

enfermagem.ubajara@ifce.edu.br

Umirim

Marta Silvanere Pereira Dantas - Enfermeira

marta.dantas@ifce.edu.br

Nathalia da Silva Costa Holanda - Técnica de Enfermagem

nathalia.holanda@ifce.edu.br

INSTITUTO FEDERAL
Ceará

LUMEN AD VIAM

PPCCLIS
PROGRAMA DE PÓS-GRADUAÇÃO
CUIDADOS CLÍNICOS EM SAÚDE
